# Molecular and Morphological Inference of Three Cryptic Species within the *Merodon aureus* Species Group (Diptera: Syrphidae)

**DOI:** 10.1371/journal.pone.0160001

**Published:** 2016-08-17

**Authors:** Ljiljana Šašić, Jelena Ačanski, Ante Vujić, Gunilla Ståhls, Snežana Radenković, Dubravka Milić, Dragana Obreht Vidaković, Mihajla Đan

**Affiliations:** 1 Department of Biology and Ecology, Faculty of Sciences, University of Novi Sad, Novi Sad, Serbia; 2 Laboratory for Biosystems Research, BioSense Institute—Research Institute for Information Technologies in Biosystems, University of Novi Sad, Novi Sad, Serbia; 3 Zoology Unit, Finnish Museum of Natural History, University of Helsinki, Helsinki, Finland; Queensland University of Technology, AUSTRALIA

## Abstract

The *Merodon aureus* species group (Diptera: Syrphidae: Eristalinae) comprises a number of different sub-groups and species complexes. In this study we focus on resolving the taxonomic status of the entity previously identified as *M*. *cinereus* B, here identified as *M*. *atratus* species complex. We used an integrative approach based on morphological descriptions, combined with supporting characters that were obtained from molecular analyses of the mitochondrial cytochrome c oxidase I gene as well as from geometric morphometry of wing and surstylus shapes and environmental niche comparisons. All applied data and methods distinguished and supported three morphologically cryptic species: *M*. *atratus* **stat. nov.**, *M*. *virgatus* **sp. nov.** and *M*. *balkanicus* **sp. nov.**, which constitute the *M*. *atratus* species complex. We present an identification key for the sub-groups and species complexes of the *M*. *aureus* species group occurring in Europe, describe the taxa and discuss the utility of the applied methods for species delimitation. The estimated divergence times for the species splits of these taxa coincide with the Pleistocene Günz-Mindel interglaciation and the Great interglaciation (between the Ris and Mindel glacial periods).

## Introduction

The hoverfly genus *Merodon* Meigen, 1803 (Diptera: Syrphidae: Eristalinae) is distributed throughout the Palaearctic and Afrotropical regions, with the highest species diversity in Eastern Europe and Asia Minor [1, 2]. The larvae are phytophagous, developing inside bulbs or rhizomes of monocotyledonous plants [3, 4]. Adults of *Merodon* species morphologically mimic bumblebees and bees (Hymenoptera: Apidae), and feed on pollen and nectar from early spring to late autumn [5]. The total number of *Merodon* species is approximately 160, and the genus recently became the most speciose European (including Turkey) hoverfly genus [4]. Recent taxonomic papers dealing with the genus *Merodon* have examined different groups of species and identified many taxa new to science (see [2, 4, 6–8]). Due to high morphological similarity, many species remain incompletely described and are thus inadequately treated in existing identification keys [5]. There is also an apparent need for a subgeneric classification, resolving taxonomic status and identification of many Palearctic *Merodon* species [2].

An integrative taxonomic approach has helped greatly in resolving outstanding taxonomical questions for several hoverfly taxa where traditional methods have proven inconclusive [7, 9, 10]. Within the genus *Merodon*, many cryptic species exhibiting minimal morphological differences have been detected in recent studies combining molecular and morphometric data to delimit species (see [4, 8, 11–14]). In molecular taxonomic studies of hoverflies, both the 3’ and 5’ ends of the mitochondrial (mtDNA) cytochrome c oxidase I (*COI*) gene are widely used (see [1, 2, 6, 7, 10, 11, 13, 15, 16]). In addition, quantification of wing and surstylus shape trait variations using geometric morphometric approach has been successfully applied in several recent taxonomic studies on hoverfly taxa. This method detects subtle wing shape differences and has provided additional evidence supporting the delimitation of cryptic species [9, 10, 12, 17, 18]. Environmental factors can also help to delimit species if we assume that each species potentially forms its own environmental niche space. Environmental niche modelling (ENM) uses widely available environmental and georeferenced distribution data [19], and it has proven useful as a tool to help estimate niche conservatism or niche divergence among related taxa [20, 21, 22, 23, 24, 25]. Several studies have shown that degrees of ecological difference constitute important evidence in distinguishing species [4, 8, 24, 26, 27, 28, 29].

**Fig 1:**
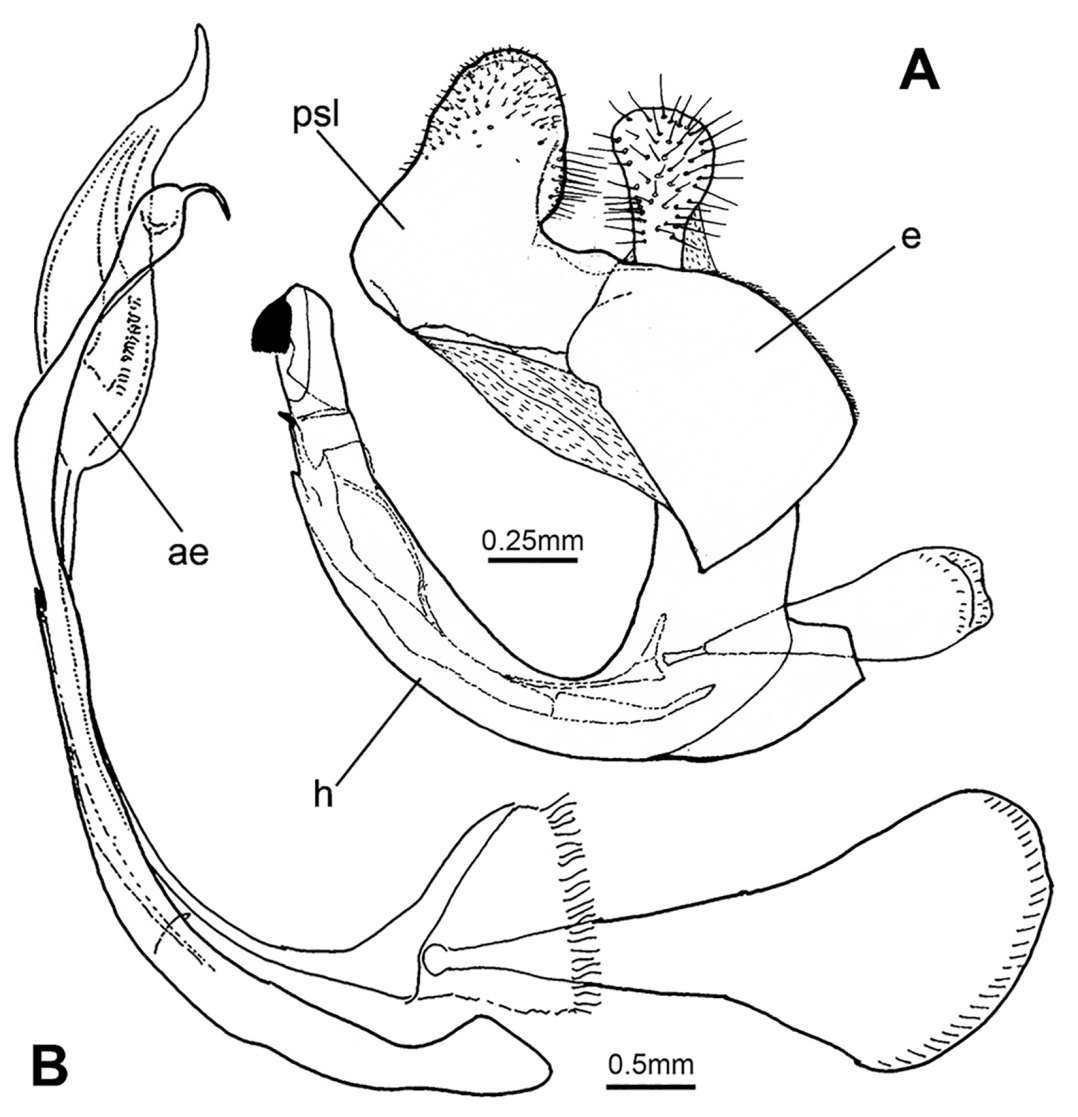
*Merodon atratus* (Oldenberg, 1919). (A) Male genitalia, lateral view (e—epandrium; h—hypandrium; psl—posterior surstylus lobe). (B) Aedeagus and associated structures (ae—aedeagus).

The *Merodon aureus* species group comprises species morphologically similar to *M*. *aureus* Fabricius, 1805. These taxa are small-sized (8–13 mm), with a short rounded abdomen, and males exhibiting a distinct spike on the metatrochanter and a characteristic structure of their genitalia, i.e. a posterior surstylus lobe with parallel margins and rounded apex and a narrow, elongated, sickle-shaped hypandrium without lateral sclerites of the aedeagus (Fig 1A and 1B). Among 114 European species listed by Speight [5] 16 share these diagnostic features (*M*. *aeneus* Megerle in Meigen, 1822, *M*. *ambiguus* Bradescu, 1986, *M*. *bessarabicus* Paramonov, 1924, *M*. *caerulescens* Loew, 1869, *M*. *chalybeus* Wiedemann in Meigen, 1822, *M*. *cinereus* (Fabricius, 1794), *M*. *dobrogensis* Bradescu, 1982, *M*. *hayati* Hurkmans in Hurkmans and Hayat, 1997, *M*. *legionensis* Marcos-García, Vujić and Mengual, 2007, *M*. *minutus* Strobl, 1893, *M*. *pumilus* Macquart in Lucas, 1849, *M*. *puniceus* Vujić, Radenković and Péres-Bañón, 2011, *M*. *quercetorum* Marcos-García, Vujić and Mengual, 2007, *M*. *sapphous* Vujić, Pérez-Bañon and Radenković, 2007, *M*. *unguicornis* Strobl in Czerny and Strobl, 1909, and *M*. *unicolor* Strobl in Czerny and Strobl, 1909).

Previous molecular and morphometric studies on *M*. *aureus* and *M*. *cinereus* indicated the presence of cryptic taxa on the Balkan Peninsula [11, 12]. Milankov et al. [11] identified six cryptic taxa and defined three of these within the *M*. *aureus* sub-group and three within the *M*. *cinereus* sub-group. The taxa within the *M*. *aureus* sub-group were named *M*. *aureus* A, B and C, respectively (as for *Merodon cinereus* A, B, C). All species of both sub-groups were diagnosed as such based on the allozyme markers employed in Milankov et al. [11]. Based on morphological characters four morphotypes could be distinguished: *M*. *aureus* A+B, *M*. *aureus* C, *M*. *cinereus* A+C, *M*. *cinereus* B. The mtDNA *COI* sequences differentiated all three *M*. *aureus* sub-group taxa from each other. *M*. *cinereus* A and *M*. *cinereus* C also differed in *COI* sequences. The *COI* sequences were identical for *M*. *aureus* C, *M*. *cinereus* B and *M*. *cinereus* C. Despite the discrepancies in the *COI* sequences and traditional morphology in separating these taxa, geometric morphometrics of wing size and shape produced phenotypic evidence congruent with the allozyme data that supported the presence of these six cryptic taxa within the *M*. *aureus* group (three in the *M*. *aureus* sub-group and three in the *M*. *cinereus* sub-group) [12]. Our ongoing studies involving recent collections in mountainous areas (Alps and on the Balkan Peninsula) have further indicated that the *Merodon cinereus* B *sensu* Milankov et al. [11] in fact represents a complex of species, and we present these results here.

The aims of the present study are to: (1) provide an identification key for the sub-groups and taxa of the European *Merodon aureus* species group; (2) define and describe the taxa within the species complex of *M*. *cinereus* B *sensu* Milankov et al. [11] (hereafter *M*. *cinereus* B); (3) integrate molecular data of mtDNA *COI*, geometric morphometrics of selected morphological structures (wings and male genitalia) and Environmental Niche Modelling for circumscription of these taxa; (4) to evaluate the power of these methods to support species delimitations; and (5) discuss the processes that led to speciation.

## Material and Methods

### Material Sampling

Specimens belonging to the *Merodon cinereus* B complex were sampled from five mountainous regions in southern Europe: the Alps (Austria), Stara Planina (Serbia), Olympus (Greece), Durmitor and Prokletije (Montenegro). Sampling was performed during the years 2011–2014. Most of the material used for molecular analyses was collected by the authors (from Austria, Alps; Montenegro, Durmitor and Prokletije; Serbia, Stara Planina). Specimens from Greece (Olympus) were donated by The Melissotheque of the Aegean, University of the Aegean, Mytilene, Greece. In addition, *M*. *aureus* group specimens deposited in the museums and universities listed below, both published and unpublished records, were studied and analyzed. The following acronyms are used in the text: BMNH—Natural History Museum, London, UK; FSUNS—University of Novi Sad, Faculty of Sciences, Department of Biology and Ecology, Serbia; MAegean—The Melissotheque of the Aegean, University of the Aegean, Mytilene, Greece; MNHN—Natural History Museum, Paris, France; MZH—Zoological Museum, Finnish Museum of Natural History, Helsinki, Finland; NMNHS—National Museum of Natural History, Sofia, Bulgaria; NHMW—Museum of Natural History, Wien, Austria; RMNH—Naturalis Biodiversity Center, Leiden, Netherlands; ZHMB—Zoological Museum of Humboldt University of Berlin, Germany; SMNS—The State Museum of Natural History, Stuttgart, Germany.

### Ethics Statement

None of the collected hoverfly species are red listed, endangered, threatened or considered to be endangered in Serbia, Montenegro, Greece or Austria. Similarly, no species collected in the present study are ranked in any IUCN list or protected by CITES. All the specimens were collected in state-owned properties. The collection of these species is not subjected to restriction by law and does not require collecting permits in these countries. Permission to collect biological specimens in protected areas was provided by the competent authorities. In Serbia our research was part of an ongoing project supported by Ministry of Environment and Spatial Planning (353-01-1345/2010-03) and Institute of Nature Protection (04-421/28.6.2010.), in Montenegro we contacted the Agency for Environment Protection (officially Agencija za zaštitu životne sredine). The Greek material was collected under a permit issued by Greek Ministry of Environment, Energy and Climate change (130276/1222).

### Taxonomic Studies

The type materials of all described European species within the *Merodon aureus* species group *sensu* Radenković et al. [13] were studied. Considerable effort was made to locate all true syntype specimens of the *Lampetia cinerea* ‘var. *atrata*’ of Oldenberg [30], finally discovered in the collection of Senckenberg German Entomological Institute, Müncheberg (SDEI).

### Nomenclatural Acts

The electronic edition of this article conforms to the requirements of the amended International Code of Zoological Nomenclature, and hence the new names contained herein are available under that Code from the electronic edition of this article. This published work and the nomenclatural acts it contains have been registered in ZooBank, the online registration system for the ICZN. The ZooBank LSIDs (Life Science Identifiers) can be resolved and the associated information viewed through any standard web browser by appending the LSID to the prefix “http://zoobank.org/”. The LSID for this publication is: urn:lsid:zoobank.org:pub:3815EE10-045E-4EE4-B915-77A52755D5BA.

The electronic edition of this work has been published in a journal with an ISSN, and has been archived and is available from the following digital repositories: PubMed Central, LOCKSS.

### Laboratory Procedures

DNA was extracted from mid and hind legs of 49 newly-collected individuals morphologically recognizable as *Merodon cinereus* B using an SDS extraction protocol [31]. Information about the examined specimens is given in S1 Table. Two regions (the 3’-end and 5’-end) of the mitochondrial *COI* gene were used in the analyses. The primers used for PCR amplification and sequencing are listed in Table 1.

**Table 1:** Primers used for amplification of *COI* gene fragments.

	Primer	Primer Sequence	Source
**3’ fragment of *COI* gene**	C1-J-2183 (alias Jerry)	5’-CAACATTTATTTTGATTTTTTGG-3’	Simon et al. [32]
	TL2-N-3014 (alias Pat)	5’-TCCAATGCACTAATCTGCCATATTA-3’	Simon et al. [32]
**5’ fragment of *COI* gene**	LCO-1490	5’-GGTCAACAAATCATAAAGATATTG-3’	Folmer et al. [33]
	HCO-2198	5’-TAAACTTCAGGGTGACCAAAAAATCA-3’	Folmer et al. [33]

Polymerase chain reactions (PCR) were carried out in 25μl reaction volumes. The reaction mixture contained 1x Taq Buffer without MgCl_2_ (ThermoScientific, Lithuania), 2mM MgCl_2_, 0.1mM of each nucleotide, 1.25U Taq polymerase (ThermoScientific, Lithuania), 5pmol of each primer, and approximately 50ng template DNA. Amplification was performed in an Eppendorf Personal Thermocycler using the following conditions for the 3’ *COI* region: initial denaturation at 95°C for 2 min; 29 cycles of denaturation at 94°C for 30 s each; 30 s annealing at 49°C; 2 min extension at 72°C; followed by a final extension of 8 min at 72°C. For the 5’ *COI* region amplification conditions were: initial denaturation at 94°C for 3 min; 29 cycles of denaturation at 94°C for 30 s each; 45 s annealing at 50°C; 1 min extension at 72°C; followed by a final extension of 8 min at 72°C. PCR products were enzymatically purified using Exonuclease I and Shrimp Alkaline Phosphatase enzymes (ThermoScientific, Lithuania) according to the manufacturer’s instructions. Sequencing was done using forward PCR primers using the BigDye Terminator v.3.1 cycle sequencing kit (Applied Biosystems) at the Sequencing Service Laboratory of the Finnish Institute for Molecular Medicine (FIMM), Helsinki, Finland.

### Molecular Analyses

DNA sequences were edited for base-calling errors using BioEdit version 7.2.5. [34]. The indel free 3’ and 5’ *COI* sequence fragments were combined in one data matrix. We used four species belonging to three subfamilies and four genera of the family Syrphidae as outgroups for tree constructions: *Merodon albifasciatus* Macquart, 1842 and *Eumerus amoenus* Loew, 1848 (Eristalinae), *Xanthogramma citrofasciatum* (De Geer, 1776) (Syrphinae) and *Archimicrodon* sp. (Microdontinae) (S1 Table). All analyses were rooted on *Archimicrodon* sp. Maximum parsimony (MP) analysis for combined *COI* sequences was performed in NONA [35] spawned with the aid of Winclada [36] using the heuristic search algorithm with 1000 random addition replicates (mult*1000), holding 100 trees per round (hold/100), maxtrees set to 100 000 and applying tree-bisection–reconnection branch swapping. Nodal support for the tree was assessed using non-parametric bootstrapping with 1000 replicates using Winclada. A Maximum Likelihood (ML) tree was constructed using RAxML 8.2.8 [37] using the CIPRES Science Gateway web portal [38] under the general time-reversible (GTR) evolutionary model with gamma distribution (GTRGAMMA) [39], and branch support was estimated with 1000 non-parametric bootstrap replicates. Combined *COI* sequences were also used for Median-joining haplotype network construction by NETWORK 5 [40]. DNA polymorphism was calculated and a haplotype data file was generated using DnaSP version 5 [41], while Arlequin 3.5.1.3 software was used for analysis of molecular variance (AMOVA) and calculations of pairwise species genetic divergence [42]. Uncorrected sequence divergence values (p distances) were calculated using MEGA version 6 [43].

Divergence time estimations were done assuming a 1.15% substitution rate per million years, which is commonly used for insects [44], and using a linear regression method (see [45]), according to the equation t = p/2r (t—divergence time, p—uncorrected p distance and r—rate of substitution per million years) and by BEAST 1.8.1 program package [46]. Xml files were generated in BEAUti version 1.8.1. As clock model we used Strict clock as well as Lognormal relaxed clock [47] and Birth-death tree model in both cases. Substitution model was Hasegawa-Kishino-Yano model (HKY). Convergence of parameter values was assessed using Tracer, version 1.6 [48]. 10^7^ generations were run, sampled every 100 generations. The output of the first 1000 trees was discarded as burn-in. The sampled posterior trees were summarized using TreeAnnotator, version 1.8.1, choosing “Maximum clade credibility tree” and “Mean heights”, and displayed in FigTree version 1.4.2 [49].

Putative species limits were explored with the Automatic Barcode Gap Discovery (ABGD) software [50] using default settings (Pmin = 0.001, Pmax = 0.1, Steps = 10, X (relative gap width) = 1.5, Nb bins = 20) and a Kimura two parameters model for pairwise distance calculation [51]. This program automatically finds the distance at which a barcode gap occurs and sorts the sequences into groups, i.e. putative species, based on this distance. This procedure is then recursively applied to the previously obtained groups of sequences until no more splits can be made [50].

### Geometric Morphometric Analysis

High-resolution photographs of the wings and surstyli were made using a Leica DFC320 video camera attached to a Leica MZ16 stereomicroscope. The landmark and semi-landmark digitalizations were carried out using the software TpsDig 2.05 [52]. Principal component analysis (PCA) was used to explore wing shape variation among the specimens. Multivariate analysis of variance (MANOVA) conducted on principal components (PC) was used to confirm that the observed variations were connected with shape differences between taxa. Canonical variate analysis (CVA) and discriminant analysis (DA) were used to test wing and surstylus shape differences between investigated species. Phenograms were generated by UPGMA clustering using Squared Mahalanobis Distances produced by DA.

Correlation between Squared Mahalanobis distances of both wing and surstylus and geographic distance was addressed using the two tailed Mantel test [53] with 10000 permutations in PaSSaGe software [54]. Geographic distance was taken as the minimum distance between two species.

#### Wing Morphometry

Geometric morphometric analysis of wing shape was conducted using a total of 63 specimens of the *Merodon cinereus* B complex (S3 Table). Due to our limited sample sizes, we analyzed males and females together. The right wing of each specimen was removed using micro-scissors and then mounted in Hoyer’s medium on a microscopic slide. All wing slides are archived and labeled with a unique code in the FSUNS collection, together with other data relevant to the specimens. Eleven homologous landmarks at vein intersections and terminations that could be reliably identified and representing wing shape were selected. Generalized least squares Procrustes superimposition (GLS) was used to minimize non-shape variations in location, scale and orientation of wings, and also to superimpose the wings in a common coordinate system [55, 56]. For the wing shape analysis, partial warp scores (thin-plate spline coefficients) were calculated [56].

GLS and partial-warp scores were computed using CoordGen 7.14 and CVAgen 7.14a, which are elements of the IMP software package [57]. MorphoJ v2.0 was used to visualize the thin-plate spline deformation [58].

#### Surstylus Morphometry

Shape analysis of the right posterior surstylus lobe (Fig 1A: psl) of male genitalia was carried out on 26 specimens of the *Merodon cinereus* B complex using a semi-landmark geometric morphometric approach (S4 Table). The right posterior surstylus lobe was removed using a scalpel and placed on its side in glycerol on a microscopic slide, with a cover slip placed on top of the surstylus to immobilize it. As the lobes of the surstylus are rounded structures without marked lateral processes or other structurally-defined points along the margin, 20 semi-landmarks were digitized along the lateral margin of the lobe (from the membranous part of the epandrium to the end of the surstylus). The software CoordGen 7.14 with an integrated Semiland module was used for semi-landmark superimposition using a distance-minimizing protocol, minimizing the shape differences due to the arbitrary nature of semi-landmark positions along the curve [56, 59].

### Environmental Niche Comparisons

To examine environmental niche divergence between the putative species of the *Merodon cinereus* B complex three types of environmental variables were used: bioclimatic, elevation and habitat. Bioclimatic variables and elevation data were obtained from WorldClim database [60]. This database has a set of climate layers representing bioclimatic variables, derived from monthly temperatures and rainfall recorded worldwide [60]. Bioclimatic and elevation variables were first tested for multicollinearity with VIF (variance inflation factors) analysis in R platform [61] using the package *usdm* [62] for all species. As VIF values showed a high level of collinearity, we sequentially dropped the covariates with the highest values, recalculated the VIFs and repeated this process until all values were smaller than 10 [63]. After evaluation, the remaining variables were used to model the current potential distribution of each investigated species (Table 2). Habitat variables were obtained from Corine Land Cover (CLC) (Corine Land Cover 2012) The standard CLC nomenclature includes 44 land cover classes. Land cover variables were transformed into different land cover categories within every grid cell in ArcView GIS 10.1. All variables have a spatial resolution of 2.5 arc minutes (approximately 5km).

**Table 2:** Bioclimatic and elevation variables selected for the environmental niche analysis among the analyzed taxa.

	*M*. *atratus*	*M*. *balkanicus* sp. nov.	*M*. *virgatus* sp. nov.
**Elevation**		x	
**BIO2**	x		x
**BIO4**		x	
**BIO6**	x	x	x
**BIO8**	x	x	x
**BIO9**	x		
**BIO13**	x		x
**BIO15**	x	x	x
**BIO18**	x		
**BIO19**		x	

Maximum entropy algorithm implemented in MAXENT software [64, 65] was used to produce continuous suitability scores for each cell (from 0 to 1) based on simulations of realized distributions. We used the default parameters of MAXENT and included 75% of species records for training and 25% for testing the model. In total, we had 85 presence records for the *M*. *cinereus* B species complex. Duplicate records were removed from the analyses.

Niche overlap, identity and similarity tests were calculated using ENM Tools software [19, 66] based on MAXENT scores. Niche overlap, measured as Schoener's D [67], ranges from 0 (no overlap) to 1 (complete overlap) for each comparison among pairs of models.

For the niche identity test, a result is obtained by testing the hypothesis that two niches are identical. This randomization test compares the overlap score from actual species occurrences with distribution of overlap scores produced by 100 pseudoreplicate datasets. Niches were considered statistically different between species if the observed value of niche overlap was less than the niche overlap value from 95 or all 100 of the niche overlap values estimated from the randomized runs (α = 0.05 and 0.01, respectively). For the similarity test, two sets of results are obtained: a comparison of niche overlaps between the observed points of taxon A and random points drawn from the background area of taxon B (A vs. B), or the converse (B vs. A). Niche similarity is viewed as a two-tailed test (α = 0.01), where the overlap of two ranges is higher or lower than expected by chance alone, with chance defined by the range of scores produced by the pseudoreplicated data.

## Results

### Taxonomy of *Merodon aureus* Species Group

**Fig 2:**
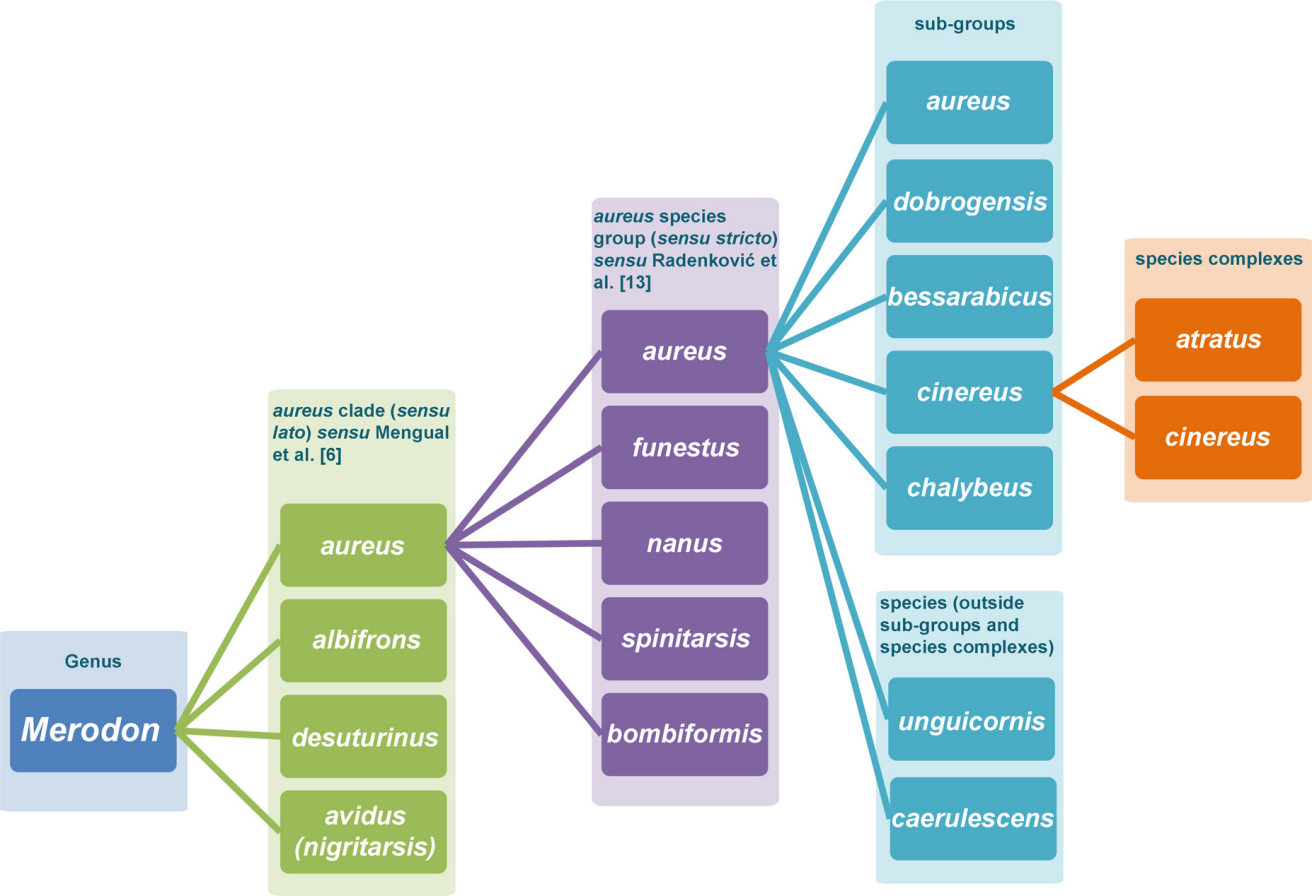
*Merodon aureus* clade *sensu* Mengual et al. [6].

As all of the taxa within the *Merodon aureus* species group show subtle morphological variation, we here distinguish different levels of morphological differentiation and propose a system of four levels (ranks) for classification of the genus *Merodon* (Fig 2). The broadest (first) level consists of large monophyletic *clades* (see Key I below) where each contains multiple morphologically different species groups. The second broadest level involves taxa that constitute of morphologically defined *species groups* within clades, such as the *M*. *aureus* and *M*. *funestus* species groups [6], or the *M*. *nanus species* group [4] (Fig 2). The third level represents *sub-groups* that based on our classification include species with very similar morphologies, but exhibiting small, consistent interspecific character variations that facilitate their distinction (see Key II bellow). Finally, the narrowest (fourth) level are *species complexes* that comprise morphologically inseparable taxa based on classical taxonomical methods, which can only be resolved by employing integrative taxonomy involving molecular markers, morphometry, and ecology.

**Table:** 

Key I	
Clades of genus *Merodon sensu* Mengual et al. [6] and Vujić et al. [2]	
1. Mid coxa without long hairs, posteriorly bare	*Merodon nigritarsis* (*avidus*) clade
- Mid coxa with at least a few long hairs posteriorly	2
2. Anterior anepisternum below postpronotum with bare area	*Merodon albifrons+desuturinus* clade *sensu* Vujić et al. [2]
–Anterior anepisternum below postpronotum with many long hairs; small species with stocky abdomen	*Merodon aureus* clade (*sensu lato*)

**Table:** 

Key II	
Taxa of the *Merodon aureus* species group *sensu* Radenković et al. [13] occurring in Central Europe and the Mediterranean region (for diagnostic features of the group, see Introduction).	
1. Tibiae and tarsi mostly pale, at least basal half of fore and mid tibiae and tarsi ventrally	2
- Tibiae and tarsi predominantly black	3
2. Tergites reddish	*Merodon dobrogensis* sub-group (Eastern Mediterranean, 3 species)
- Tergites dark, exceptionally with small orange spots on lateral sides of tergites 2-3	*Merodon bessarabicus* sub-group (central and southern Europe, 6 species)
3. Tergites 2–3 with pale lateral spots	*Merodon unguicornis* (Western Mediterranean)
- Tergites uniformly dark	4
4. Mesonotum with only pale pile	*Merodon aureus* sub-group (central and southern Europe, 3 species)
- Mesonotum at least near wing base with black pile	5
5. Species with strong blue body lustre	*Merodon caerulescens* (Aegean islands)
- Species with dark brown or greenish body lustre	6
6. Body pile shorter; pile on scutellum in male shorter than hind basitarsus; tergites in female without microtrichose bands, or with small ones on tergites 2-3	*Merodon chalybeus* sub-group (islands, coastal zone or low altitudes in Mediterranean, 4 species)
- Body pile longer; pile on scutellum in male as long as or longer than hind basitarsus; tergites 2-4 in female with pair of microtrichose stripes	*Merodon cinereus* sub-group (Central and South European mountains, 2 species complexes).

### Morphological Differentiation and Integrative Taxonomy

**Fig 3:**
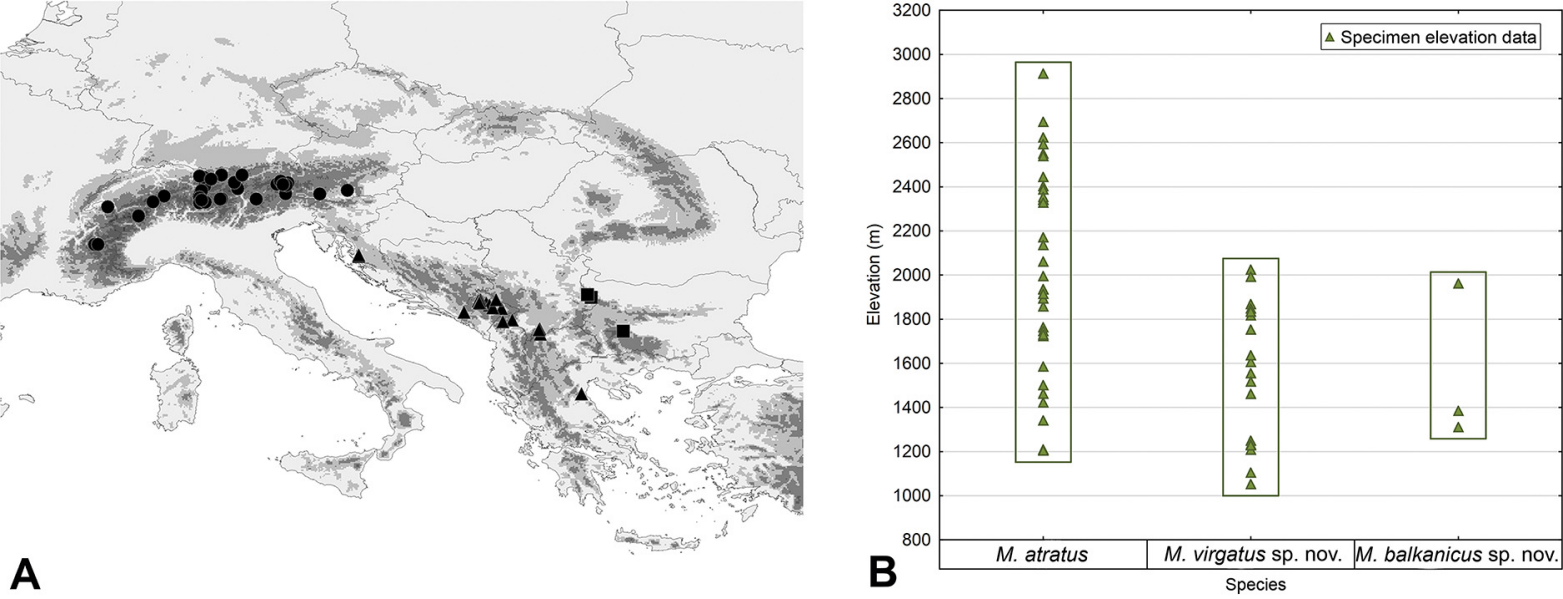
The distributional and elevation ranges of the *Merodon atratus* species complex. (A) Map of Europe showing the distribution of the three species of the *M*. *atratus* species complex and sampling sites. The circles **●** stand for *M*. *atratus*, triangles ▲ for *M*. *virgatus* **sp. nov.**, and squares ■ for *M*. *balkanicus* **sp. nov.** (B) Variability plot of elevation ranges of taxa of the *M*. *atratus* species complex.

**Fig 4:**
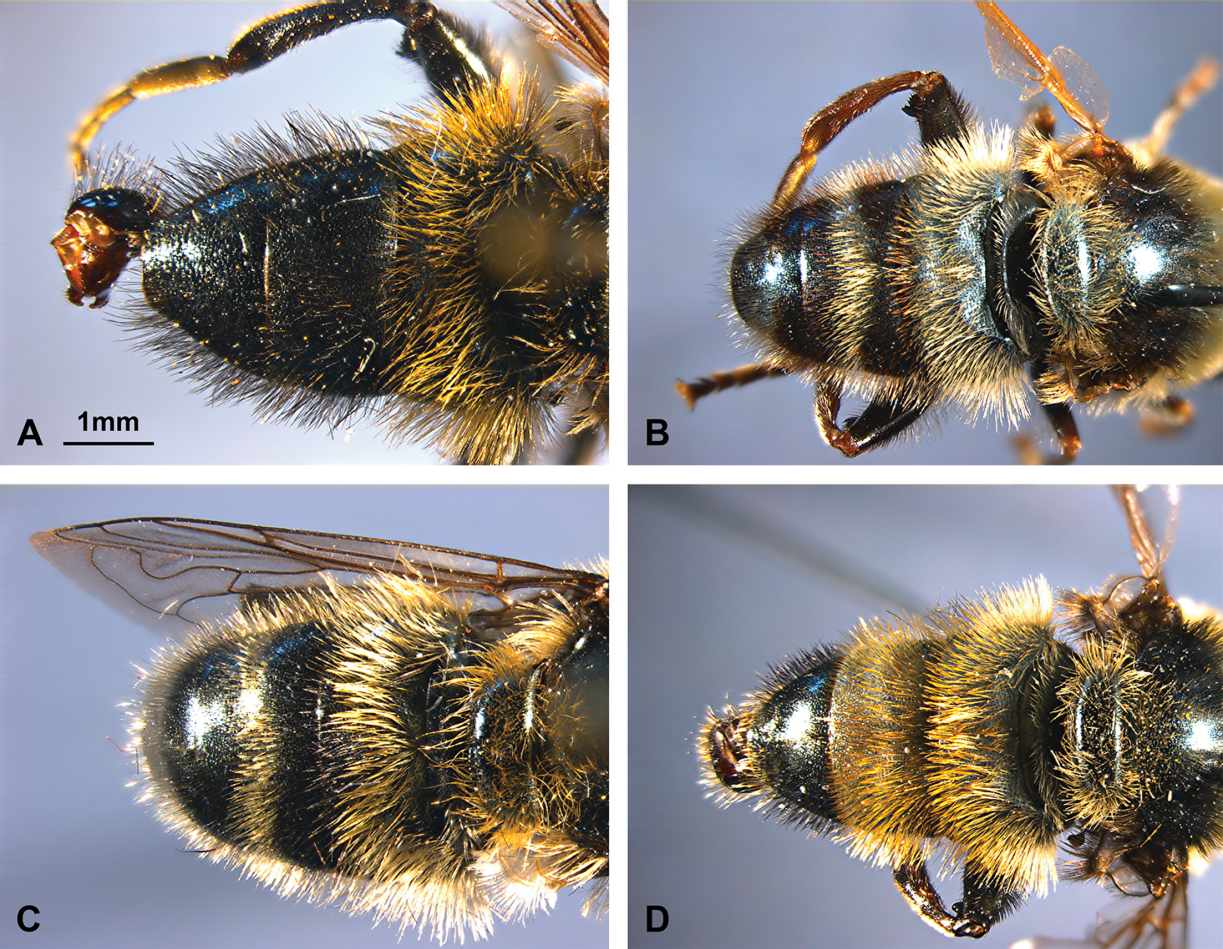
Abdomen, males, dorsal view. (A) *Merodon atratus*; (B) *M*. *virgatus* **sp. nov.**; (C) *M*. *balkanicus* **sp. nov.**; (D) *M*. *cinereus*.

The taxon *Merodon atratus*, previously published as *M*. *cinereus* B in Milankov et al. [11], belongs to the *M*. *aureus* clade in the sense of Mengual et al. [6] and the *M*. *aureus* species group according to Radenković et al. [13]. A taxonomic study of material from different collections throughout the geographic range of *M*. *atratus* (Fig 3A) in Europe indicated the existence of consistent morphological differences between populations in different geographical regions, especially between the Alps and the Balkan mountains. The main differences between populations from the Alps and the Balkan Peninsula are the quantity and arrangement of the black pile on the mesoscutum and tergites. In Alpine populations, the mesoscutum and tergites 2–4 can be almost entirely covered with black pile (Fig 4A). In populations from the mountains of the Balkan Peninsula, the black pile on the mesoscutum is reduced to a stripe between the wing bases (Fig 4B) and stripes of black pile on tergites 2–4 (Fig 4B and 4C). Based on these morphological differences, we concluded that *M*. *atratus* is a complex of species, and we applied an integrative taxonomic approach to prove this.

### Molecular Analyses

**Fig 5:**
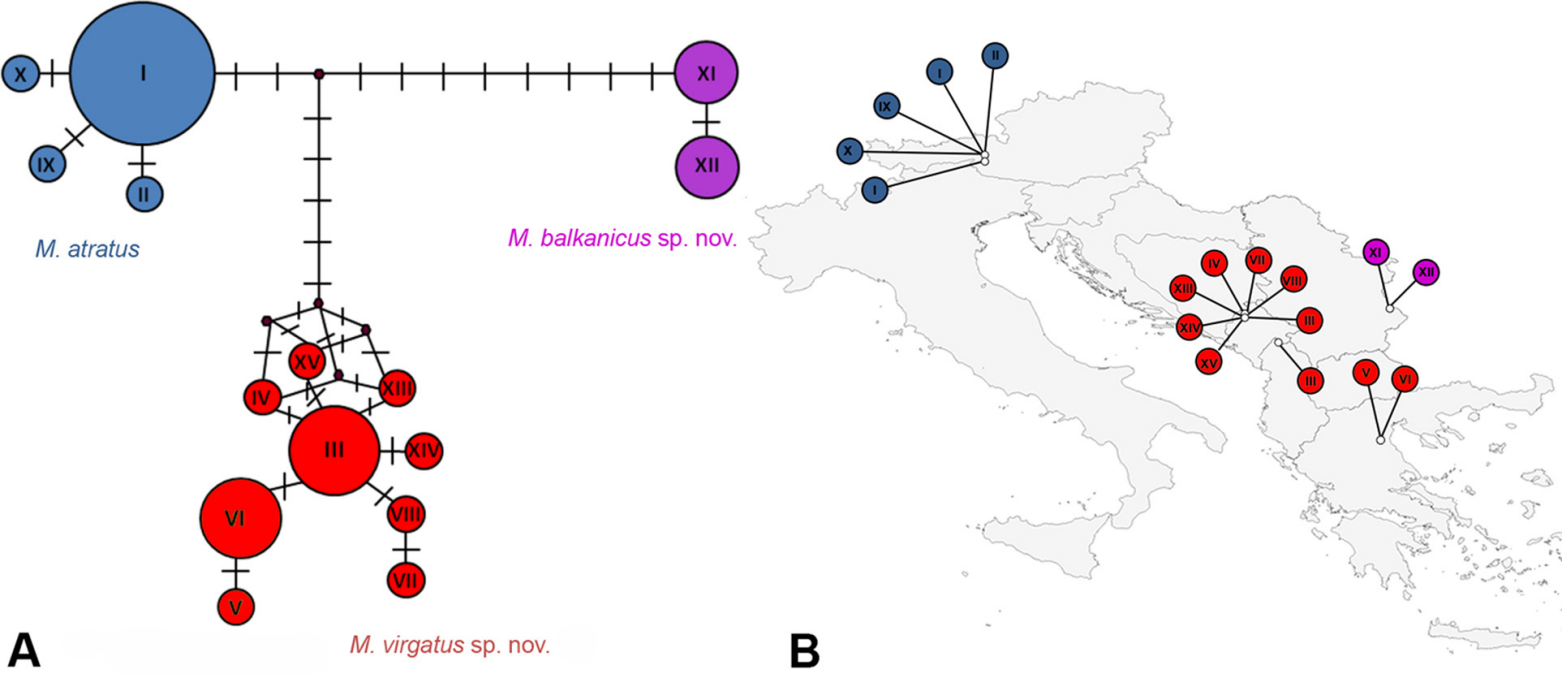
Haplotype diversity within the *Merodon atratus* species complex. (A) Haplotype Median-joining network; vertical lines represent the number of mutational steps. (B) Map of haplotype distribution.

In total, 49 specimens from the *Merodon atratus* species complex were included in molecular analyses. Amplification of both the 3’-end and 5’-end of the *COI* gene was successful for all individuals. The combined dataset of the *COI* gene comprised 49 sequences, with a total length of 1313 nucleotide characters. The sequences generated 15 haplotypes, of which four represented *M*. *atratus* (I, II, IX, X), nine represented *M*. *virgatus* **sp. nov.** (IV, VII, VIII, XIII, XIV, XV corresponding to the Durmitor population, III corresponded to the Prokletije specimen, and V and VI corresponding to the Olympus population), and two represented *M*. *balkanicus* **sp. nov.** (XI, XII) (Fig 5A and S2 Table). There were no shared haplotypes among species within the *M*. *atratus* species complex. The Median-joining network of *M*. *atratus* species complex haplotypes and a corresponding distributional map are shown in Fig 5A and 5B. Haplotype diversity (Hd) was 0.838 (± 0.038), nucleotide diversity (Pi) was 0.00625 and the average number of nucleotide differences (K) was 8.204. AMOVA indicated a high level of interspecific variation (fixation index, Фst = 0.94061) within the species complex and, according to Фst values, interspecific variation was highly significant (p<0.01) for all species pairs. The uncorrected pairwise divergences (p) between the species of the *M*. *atratus* species complex were 0.8% (*M*. *atratus* vs. *M*. *virgatus* **sp. nov.**), 0.9% (*M*. *atratus* vs. *M*. *balkanicus* **sp. nov.**) and 1.4% (*M*. *virgatus* **sp. nov.** vs. *M*. *balkanicus* **sp. nov.**).

**Fig 6:**
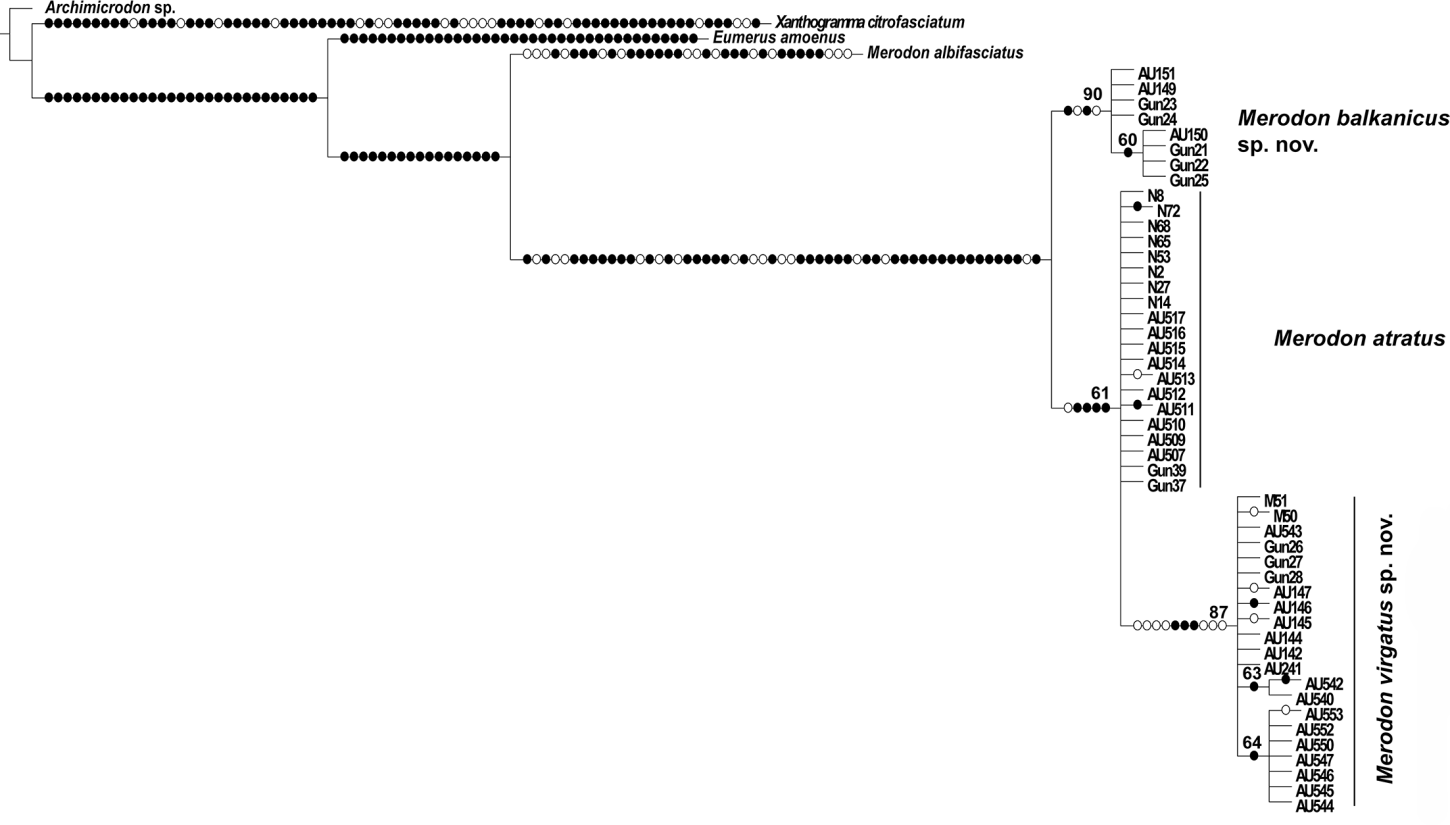
Strict consensus tree based on 3 equally parsimonious trees from analysis of combined *COI* sequences. Length 526 steps, Consistency index (CI) 81, Retention index (RI) 82. Bootstrap values ≥60 are indicated near nodes. Filled circles represent non-homoplasyous changes and open circles homoplasyous changes.

The combined sequences of 3'-end and 5'-end of *COI* were used for MP and ML tree constructions. Parsimony analysis resulted in 3 equally parsimonious trees of 525 steps in length (Consistency Index = 81, Retention Index = 83). The parsimony strict consensus tree (length = 526) is shown in Fig 6. According to MP tree topology *M*. *balkanicus* **sp. nov.** forms one clade and *M*. *virgatus* **sp. nov.** is nested within *M*. *atratus* clade, while on ML tree they form separate clades (S1 Fig).

#### Automatic Barcode Gap Discovery (ABGD)

The ABGD method was run with a prior maximum divergence of intraspecific diversity, i.e. species divergence from 0.001 to 0.1. Combined sequences of two *COI* fragments were analyzed for the determination of the ‘barcoding gap’ between taxa of the *Merodon atratus* species complex. The number of groups for the recursive partition was three, with prior divergences of 0.0046, 0.0028, 0.0017 and 0.0010. The primary partition was concordant with the recursive for all of the prior divergence values. The three partition groups correspond to the three taxa of the *M*. *atratus* species complex.

#### Estimation of Divergence Time

Sequence divergences (uncorrected p distances) of the *COI* gene were used for assessment of relative divergence times between taxa within the *Merodon atratus* species complex by a linear-regression method. *M*. *balkanicus* **sp. nov.** separated from *M*. *atratus*+*M*. *virgatus* **sp. nov.** around 492.3 ka BP (thousand years Before Present) (p = 1.1%), while the estimated split between *M*. *atratus* and *M*. *virgatus* **sp. nov.** took place 350.3 ka BP (p = 1%). The divergence time coincides with the periods of the Günz-Mindel interglaciation and the Great interglaciation between the Mindel and Ris periods, during the Pleistocene. According to Milankovitch [68], the Mindel glaciation took place during the period from 480 ka BP to 434 ka BP, and the interval separating the Mindel from Ris periods begun around 434 ka BP and finished around 232 ka BP [69].

**Fig 7:**
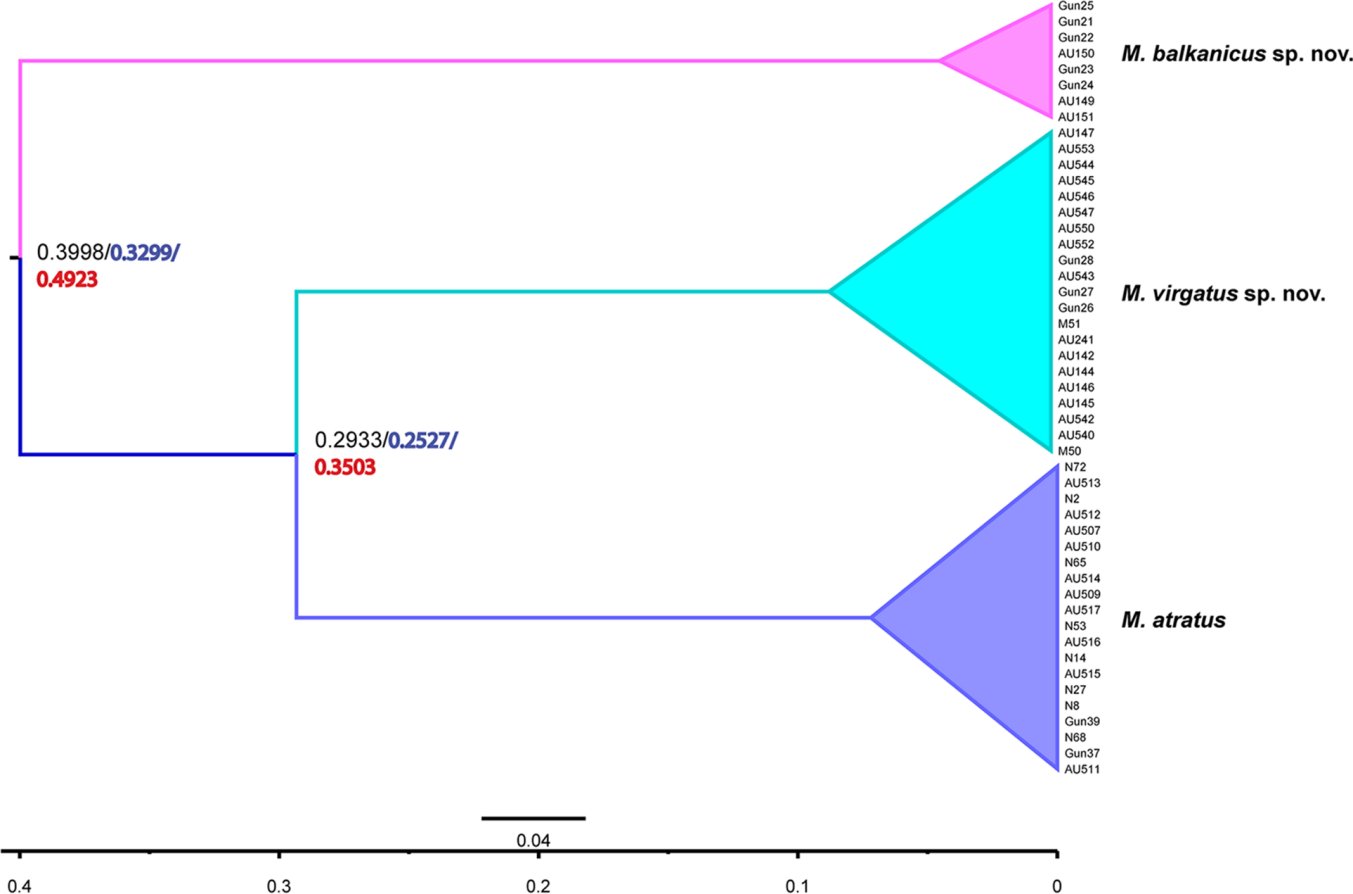
Species tree chronogram of *Merodon atratus* species complex inferred using BEAST. Mean node ages were estimated using a Strict clock model (substitution rate 1.15% per million years) and Birth-Death tree model (black numbers). The scale bar represents million years. The time axis (mean ages) is indicated at the bottom. The blue numbers represent mean node ages estimated using Lognormal relaxed clock model [47] and Birth-Death tree model and the red numbers represent divergence time estimated by linear regression method [45].

Divergence time estimated using a Strict clock model (399.8 ka BP and 293.3ka BP) and Lognormal relaxed clock model (329.9 ka BP and 252.7 ka BP) differed from the linear regression method estimates and coincides with Great interglaciation between the Mindel and Ris periods. Species tree chronogram of *Merodon atratus* species complex is presented in Fig 7.

### Geometric Morphometric Evidence

#### Wing Shape

Based on morphological and molecular data, specimens of the *Merodon atratus* species complex could be separated into three groups corresponding to *M*. *atratus*, *M*. *balkanicus* **sp. nov.** and *M*. *virgatus* **sp. nov.** Wing shape variations of these defined groups were studied using PCA, which produced seven PCs with an eigenvalue greater than 1. ANOVA conducted on factor scores showed that the observed variations were linked to shape differences among specimen groups in four PCs (S5 Table).

**Fig 8:**
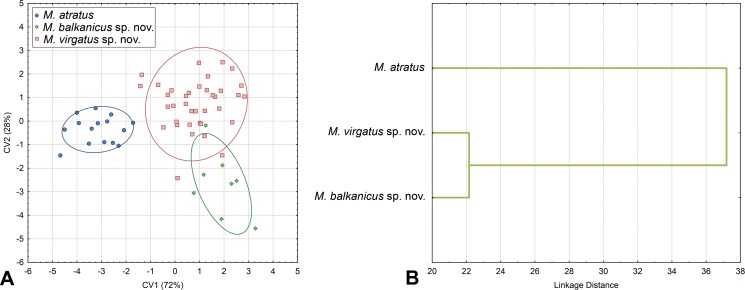
Wing shape differences among species of the *Merodon atratus* species complex. (A) Scatter plot of individual scores of CV1 and CV2. (B) UPGMA phenogram constructed using Squared Mahalanobis Distances.

**Fig 9:**
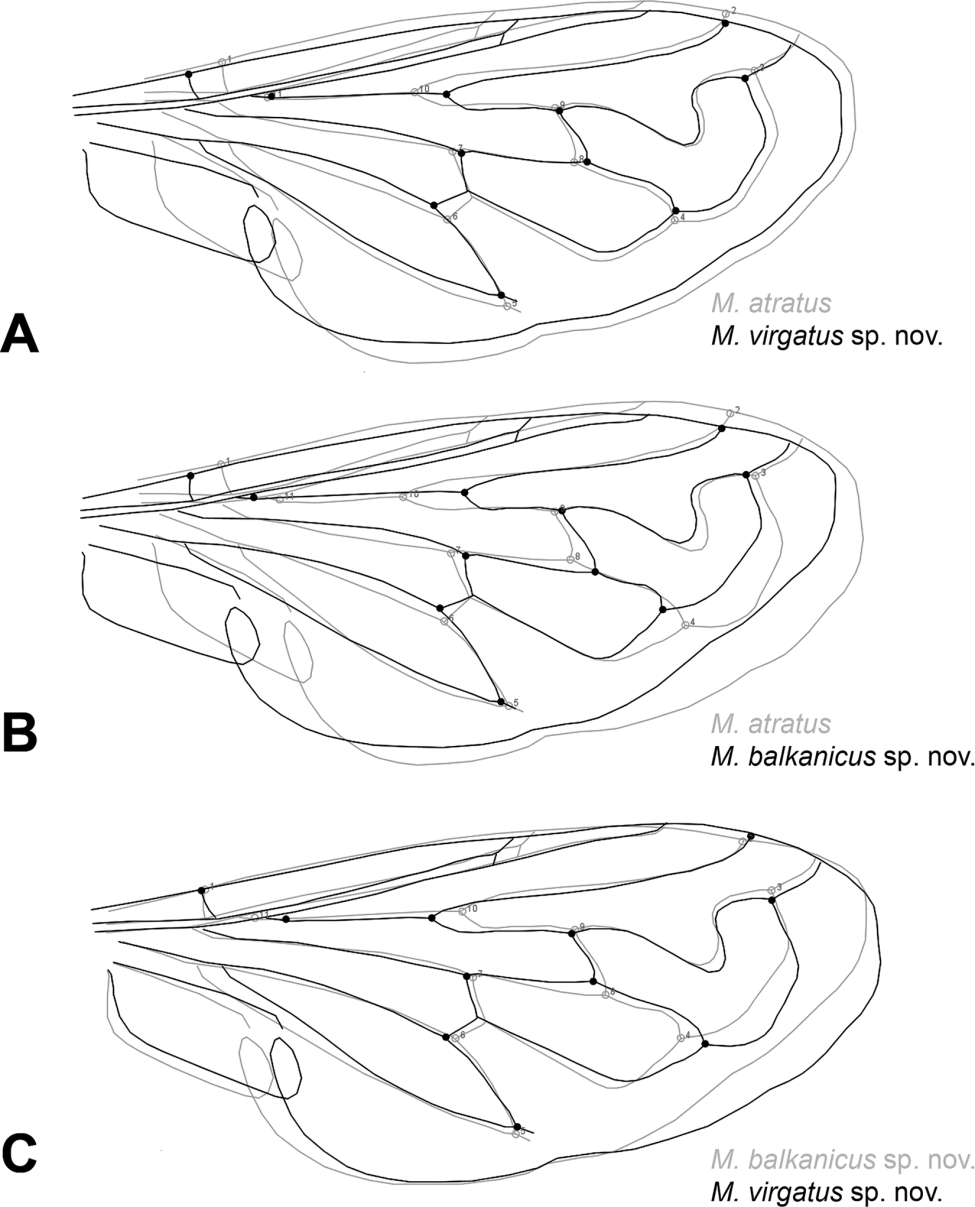
Superimposed outline drawings showing wing shape differences between analyzed species. Differences between the species were exaggerated five-fold to make them more visible.

DA correctly classified specimens with overall classification success of 95%. All specimens belonging to *M*. *atratus* were correctly classified. Only two specimens of *M*. *virgatus* **sp. nov.** grouped as *M*. *balkanicus* **sp. nov.** and one *M*. *balkanicus* **sp. nov.** as *M*. *virgatus* **sp. nov.** DA also showed that all species differs significantly by wing shape (p<0.01) (Fig 8A). Canonical analysis produced two canonical axis which are related with wing shape differences between species (CV1: Wilks’ Lambda = 0.087157; χ^2^ = 120.7824; p<0.01; CV2: Wilks’ Lambda = 0.409447; χ^2^ = 44.2009; p<0.01). CV1 described 72% of total wing shape variation and separate *M*. *atratus* from *M*. *virgatus* **sp. nov.** and *M*. *balkanicus* **sp. nov.**, while CV2 separate *M*. *virgatus* **sp. nov.** and *M*. *balkanicus* **sp. nov.** with 28% of shape variation (Fig 8A). The phenogram derived by clustering of Squared Mahalanobis distances of the discrimination analysis using UPGMA showed that *M*. *atratus* has the most distinct wing shape (Fig 8B). This species has a wider wing than the other two (Fig 9A and 9B), while the main shape differences between *M*. *virgatus* **sp. nov.** and *M*. *balkanicus* **sp. nov.** are connected with width of apical part of the wing (Fig 9C).

Mantel tests revealed that geographic distance exhibited no association with wing shape distance among investigated groups (r = 0.90529, p = 0.18740).

#### Surstylus Shape

**Fig 10:**
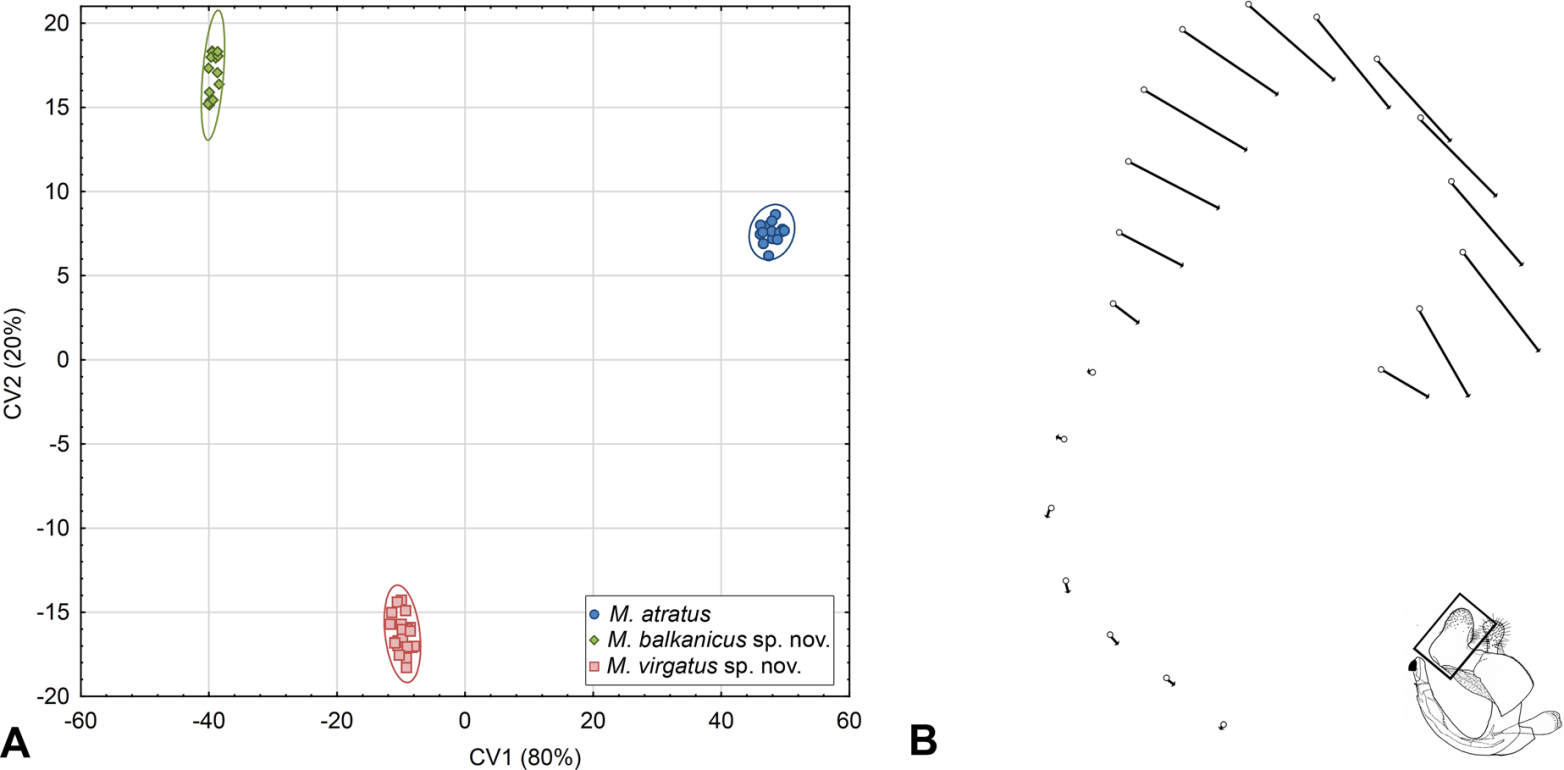
Scatter plots of surstylus shape differences among species of the *Merodon atratus* species complex. (A) Scatter plot of individual scores of CV1 and CV2. (B) Thin-plate spline deformation grids showing overall differences in posterior surstylus lobe shape between analyzed species.

For geometric morphometry analyses of surstylus shape, we adopted the same specimen groupings as for wing shape analyses, i.e. the three groups corresponding to *Merodon atratus*, *M*. *balkanicus* **sp. nov.** and *M*. *virgatus* **sp. nov.** DA showed that species within *M*. *atratus* differ highly significantly in surstylus shape (p<0.01). All specimens were correctly classified (100%) to *a priori* defined groups. CVA found two canonical axes that clearly separated all investigated specimen groups based on differences in surstylus shape (CV1: Wilks’ Lambda = 0.000004; χ^2^ = 381.7232; *p*<0.01; CV2: Wilks’ Lambda = 0.004535; χ^2^ = 164.5751; *p*<0.01). CV1 depicted the greatest differences, between *M*. *atratus* and Balkan species *M*. *balkanicus* **sp. nov.** and *M*. *virgatus* **sp. nov.**, while CV2 clearly separated *M*. *balkanicus* **sp. nov.** from *M*. *virgatus* **sp. nov.** (Fig 10A).

**Fig 11:**
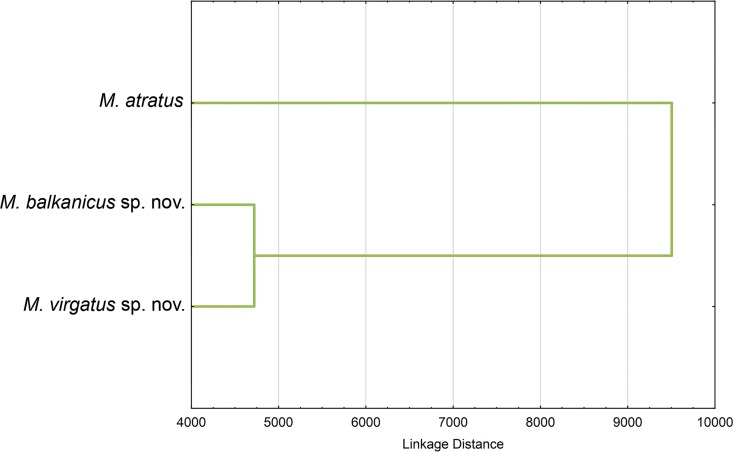
Surstylus shape differences among species of the *Merodon atratus* species complex. UPGMA phenogram constructed using Squared Mahalanobis Distances.

Based on the Squared Mahalanobis Distances produced by DA, the most similar surstylus shapes were between *M*. *virgatus* **sp. nov.** and *M*. *balkanicus* **sp. nov.**, while *M*. *atratus* had the most distinctive shape (Fig 11). Overall, differences in surstylus shape among investigated specimen groups are mostly connected with the posterior part of the posterior surstylus lobe (Fig 10B).

Mantel tests showed that geographical distribution has no impact on morphological differentiation of surstylus among investigated groups (r = 0.93995, p = 0.18730).

### Environmental Niche Comparisons

Niche overlap among the investigated species of the *Merodon atratus* species complex was generally low, ranging from 0.213 for *M*. *balkanicus* **sp. nov.**—*M*. *virgatus* **sp. nov.** to 0.328 for *M*. *atratus—M*. *balkanicus* **sp. nov.** (Table 3).

**Table 3:** Environmental niche comparisons for the species of the *Merodon atratus* species complex. Niche overlap values are presented for the comparisons of niche identity and similarity of species A with species B.

*Merodon* species		Niche overlap (Schoener's D index)	Identity test (±SD)	Niche similarity test (±SD)	
A	B			A vs. B	B vs. A
*M*. *atratus*	*M*. *balkanicus* **sp. nov.**	0.328	0.544±0.036**	0.159±0.029**	0.140±0.012**
*M*. *atratus*	*M*. *virgatus* **sp. nov.**	0.279	0.546±0.165*	0.121±0.096**	0.125±0.014**
*M*. *balkanicus* **sp. nov.**	*M*. *virgatus* **sp. nov.**	0.213	0.362±0.057*	0.273±0.019*	0.186±0.041*

**Environmental niches are significantly (*p≤ 0.05, **p≤ 0.01) more different or less similar than expected by chance.

SD—standard deviation.

The results of the tests of niche identity and niche similarity are presented in Table 3. In both cases, the null hypothesis of niche similarity is rejected. Randomization tests of niche identity indicated that species in each pair are more different than expected, thus they are not ecologically equivalent (p≤0.05, p≤0.01). Results of background tests also supported ecological differentiation between species pairs. In all investigated pairs, niche similarity was less similar than expected by chance. The environmental niche of *Merodon balkanicus* **sp. nov.** was less similar than expected by chance to the one of *M*. *virgatus ***sp. nov.** in both directions (Table 3). Other species pairs of *M*. *atratus* species complex shared niche spaces that were more similar than expected by chance.

### Integrative Taxonomy

In this study we describe morphological, morphometric and molecular evidence that support the presence of three independent species: *Merodon atratus*, *Merodon balkanicus* **sp. nov.** and *Merodon virgatus* **sp. nov.** (corresponding to the taxon *M*. *cinereus* B *sensu* Milankov et al. [11]). *M*. *atratus* is widespread in the Alps while *M*. *balkanicus* **sp. nov.** and *M*. *virgatus* **sp. nov.** are present on Balkan mountains.

### *Merodon atratus* Species Complex: Differential Diagnosis

The *Merodon cinereus* sub-group comprises two morphologically separable species complexes, i.e. the *M*. *cinereus* species complex and the *M*. *atratus* species complex. Members of the *M*. *atratus* species complex can be identified by having tergites 2–4 solely with black pile or with stripes of black pile (Fig 4A–4C), while in the *M*. *cinereus* species complex tergites 2–3 only have pale pile (Fig 4D).

### Species Re-Description and Description of New Species

#### Genus *Merodon* Meigen, 1803

*Merodon atratus* (Oldenberg, 1919) **stat. nov.**

Oldenberg [30] described the variety *atrata* of *Lampetia cinerea* based on three males. The type specimens of this and the taxon named as *M*. *cinereus* B in Milankov et al. [11] share identical diagnostic characters that discern them from *M*. *cinereus*. We have re-evaluated the synonyms of M. cinereus, and here elevate the taxon M. atratus stat. nov. to the rank of species.

**Fig 12:**
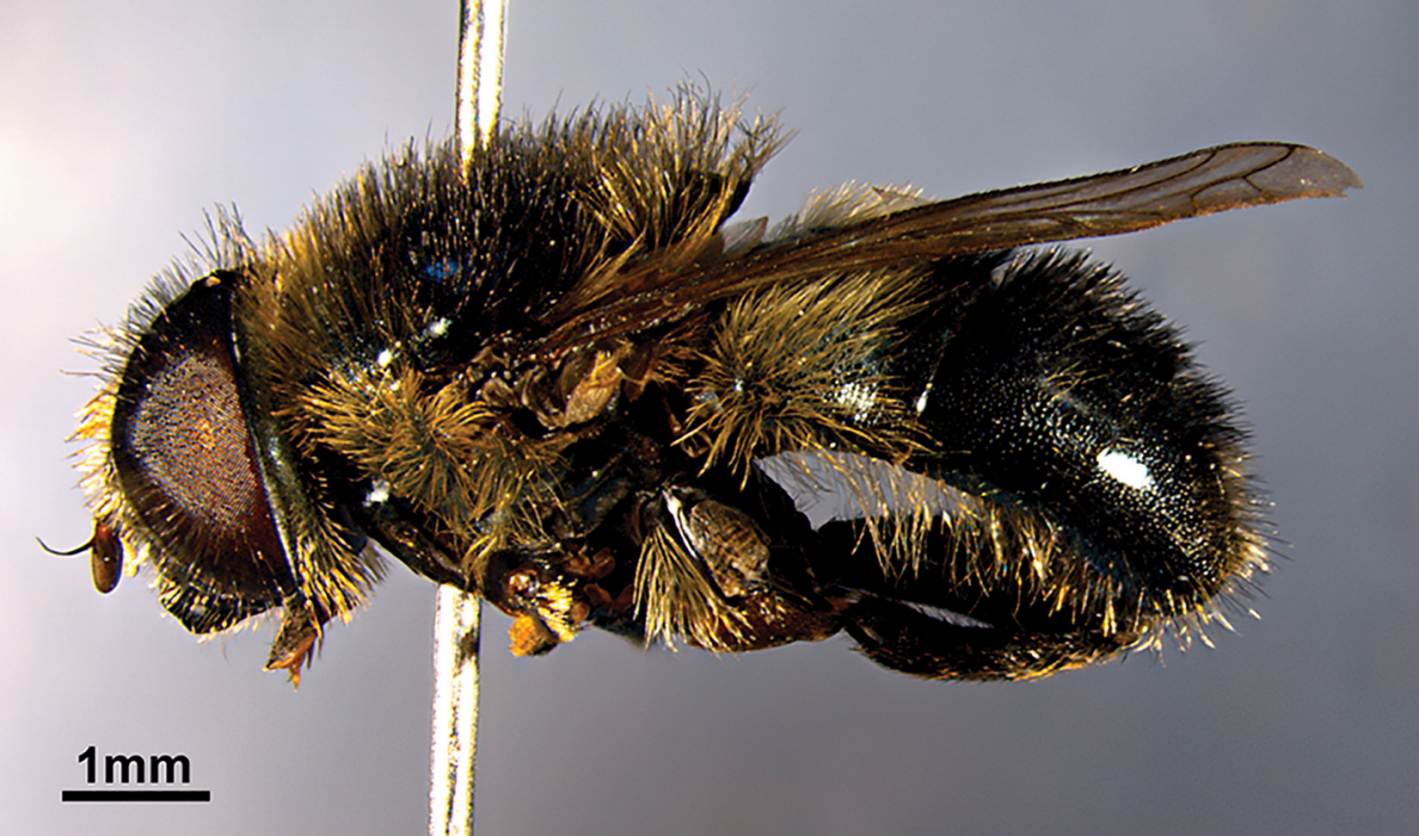
*Merodon atratus*, male, lateral view.

Diagnosis. Belongs to the *Merodon cinereus* sub-group with predominantly black pile on the mesonotum and tergites 2–4 completely covered with black pile or, in individual specimens, with stripes of black pile (Figs 4A and 12).

Type material (deposited in SDEI). Three syntypes from Switzerland: ♂ from “St. Moritz” (designated as lectotype here) and 2♂♂ from “Seiser Alp” (designated as paralectotypes). Additional material (see Appendix).

Description. Male (Fig 12).

Head. The antennae are brown to reddish-brown; basoflagellomere reddish, between 1.3 and 1.5 times longer than pedicel, dorsal margin concave between the arista and the apex, apex acute; arista reddish-brown and as long as pedicel and basoflagellomere together. Face and frons shiny black and covered with long whitish-yellow pile. Oral margin bare and lustrous black. Vertical triangle isosceles, as long as eye contiguity, shiny black and covered in long black pile. Eye contiguity about 12 ommatidia long. Ocellar triangle equilateral. Eye pile long, black over the entire surface, except for a few pale pile at the central part. Occiput covered with whitish dusting and whitish pile with some black pile intermixed.

**Fig 13:**
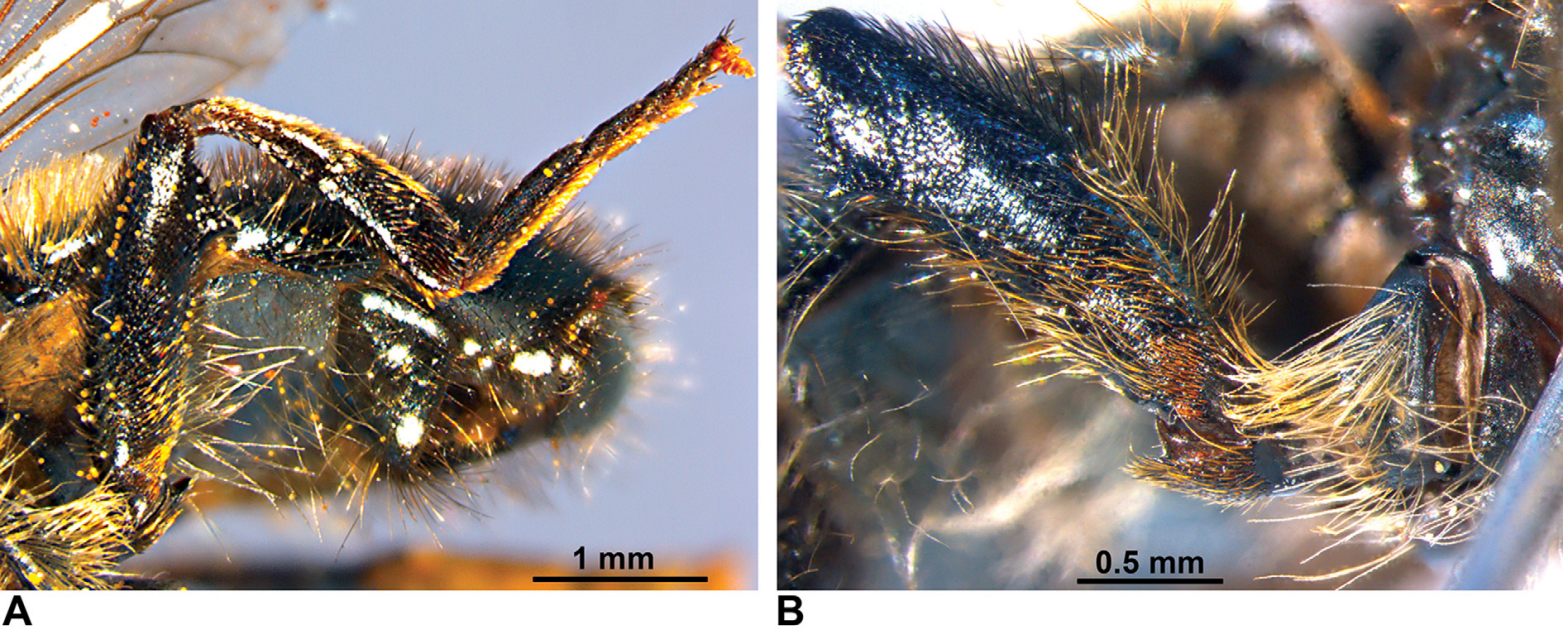
**(A) *Merodon atratus*, hind leg. (B) Hind femur and hind trochanter with an inner spike**.

Thorax. Mesonotum dark green with metallic bluish reflections, covered in long, dense, erect black and whitish pile; black pile usually concentrated in the area between wing bases (Fig 12); mesoscutum with three weak longitudinal stripes of golden dusting in anterior half. Posterior anepisternum, anepimeron and dorsal part of katepisternum with long whitish-yellow pile. Wing pale greyish, with brown veins. Dorsal and ventral calypters pale grayish. Haltere with pale brown pedicel and dark brown capitulum. Femora black; fore and mid femur covered posteriorly with long pale yellow pile and both dorsally and anteriorly with short black pile. Hind femur with long pale yellow pile basally, and with many black pile on the apical half (Fig 13A and 13B). All tibiae and tarsi black, except base and top of tibiae and tarsi ventrally which are paler; covered in yellow pile with some intermingled black ones (Fig 13A). Hind trochanter with an inner spike ending in two angular points (Fig 13B).

Abdomen. Oval, slightly longer than mesonotum; black with blue metallic reflections. Tergites 2–4 black usually without distinct white transverse bands of dusting interrupted in the middle, except in some specimens on tergite 3. Tergite 1 and anterior two-thirds of tergite 2 with pale pile, rest of tergites completely covered with black pile or with bands of black pile alternating with bands of pale ones. Sternites shiny black covered with long pale yellow pile.

Male terminalia (Fig 1). Similar to all other species of the *aureus* group. Anterior lobe of the surstylus is undeveloped with a straight ventral margin (Fig 1A); posterior surstylar lobe is rounded at the apex with long pile and parallel margins (Fig 1A: psl). Cercus elongate, without prominences. Hypandrium narrow, elongate and sickle-shaped; lateral sclerite of aedeagus reduced (Fig 1B).

Female. Similar to the male except for normal sexual dimorphism and in the following characteristics: frons shiny and covered by whitish-yellowish pile. Vertex with black pile anterior to and at the level of the ocellar triangle. Hind trochanter without a spike. 4th tarsomere of fore and mid leg and apical two tarsomeres of hind leg darkened. Abdomen shiny black, with a pair of white bands of dusting on each of tergites 2–4. On tergite 2 these bands are subparallel to the anterior margin of the tergite, whereas on tergites 3 and 4 these bands are oblique.

Variability. Eyes–color of pile varies from all black to only dark brown pile in upper quarter, rest pale; hind femur–with black pile only on apical third in some specimens, but in others with almost completely black pilosity; tergites–covered with a differing arrangement of black and pale pile.

Length. Body 7.5–9 mm; wing 5–6 mm.

*Merodon virgatus* Vujić et Radenković **sp. nov.**

urn:lsid:zoobank.org:act:61E12C6F-8A5E-4B51-9D0D-1328814543B4

*Merodon cinereus* B in Milankov et al. [11]

Diagnosis. Species with alternating stripes of pile on tergites 2–4 (Fig 4B) (in *M*. *atratus* all tergites after anterior two-thirds of tergite 2 covered with black pile, Fig 4A); eyes with black pile in upper and lower part and pale centrally (in *M*. *atratus* covered mostly with black pile); metafemur with black pile on apical third (in *M*. *atratus* from apical half to almost entirely with black pile).

Range. Mountainous species, distributed on mountain ranges near the Adriatic coast (Croatia: Velebit; Montenegro: Durmitor and Prokletije) and in the southern part of the Balkan Peninsula (FYR Macedonia and Serbia: Šar-planina; Greece: Olympus). According to available data, elevation range is from 1000 to 2010 m (Fig 3B).

Holotype. MONTENEGRO, Durmitor, Prutaš 27.7.2011. ♂ leg. Vujić (FSUNS M74).

Paratypes (see Appendix).

Etymology. The latin adjective *virgatus* refers to the morphological character of alternating stripes of pale and black pile on the mesonotum.

*Merodon balkanicus* Šašić, Ačanski et Vujić **sp. nov.**

urn:lsid:zoobank.org:act:AA4C487A-FF63-4FA6-ADA7-D0882D5755D8

Diagnosis. Very similar to *Merodon virgatus* **sp. nov.** from which it differs based on *COI* sequence divergence, wing and surstylus morphometry, distribution pattern (central part of the Balkan Peninsula, whereas *M*. *virgatus* **sp. nov.** is distributed on mountain ranges near the Adriatic coast and in the southern part of the Balkan Peninsula), and environmental niches.

Range. Endemic to Stara Planina (part of the Balkan mountain range). According to available data, the species occurs at an elevation of approximately 1300 meters (Fig 3B).

Holotype. SERBIA: Stara Planina, “Babin zub”, 11.7.2011. ♂ leg. Vujić (FSUNS L96). Paratypes (See Appendix).

Etymology. The word *balkanicus* refers to the Balkan (Stara Planina) mountain range (Eastern Serbia and Bulgaria) where the type locality of the species is.

## Discussion

### Taxonomy

The *Merodon aureus* species group contains 16 previously-known and newly-discovered taxa from the Mediterranean region and southern European mountain regions [5, 11, 13, 70, 71]. These species are classified into 5 sub-groups and two additional species (*M*. *unguicornis* and *M*. *caerulescens*) (Fig 2). The *M*. *bessarabicus* sub-group includes species with predominantly yellow tibiae and dark tergites (including *M*. *ambiguus*, *M*. *bessarabicus*, *M*. *legionensis*, *M*. *hayati*, *M*. *quercetorum*, *M*. *sapphous*); the *M*. *dobrogensis* sub-group consists of species with yellow tibiae, short body pile and red tergites (*M*. *dobrogensis* and *M*. *puniceus*); the *aureus* sub-group includes species with mostly dark tibiae and pale pile on the mesonotum (*M*. *aureus*, *M*. *pumilus*, *M*. *unicolor*). The *M*. *chalybeus* species complex (*M*. *chalybeus* and *M*. *minutus*), together with *M*. *unguicornis* and *M*. *caerulescens*, have dark tibiae. The *M*. *cinereus* sub-group (the *M*. *cinereus* and *M*. *atratus* species complexes) comprises taxa having predominantly dark tibiae, with the apical half or more of the hind tibia covered with black pile and a band of black pile between the wing bases.

### *Merodon atratus* Species Complex: Morphological and Molecular Evidence

The taxonomic challenge posed by cryptic species has been recognized for a long time, but the advent of relatively inexpensive and rapid DNA sequencing has emerged as an important tool for detecting and differentiating morphologically similar species [72]. A limited set of useful genetic markers have been used in the integrative taxonomy of closely related hoverflies. One of the most widely used molecular marker is the *COI* gene, and molecular evidence based on *COI* sequence divergences proved helpful in separating the taxa of the *Merodon atratus* species complex, as in multiple other studies (see [1, 7, 10, 13, 15]). Molecular results indicated divergence rates between 0.8–1.4% among species from *M*. *atratus* species complex. It could be argued that this level of divergences is too low to be interpreted as species level, as it is lower than the suggested 2% barcoding gap [73]. However there are cases in which two morphologically different insect species share an identical *COI* haplotype (see [1, 6, 74], or morphologically well-defined species that express an intraspecific divergence level that exceeds the interspecies level of divergence of the group (see [75]. Thus, a standard percent of species divergence cannot be generally defined but should be elucidated separately for each species group in light of other taxonomic evidence. Burns et al. [76] showed that morphologically and ecologically distinct species of skipper butterflies (Hesperiidae) could be distinguished based on 1–3 “diagnostic” nucleotides and that a divergence degree point below which individuals should be considered conspecific is unrealistic. Species delimitation based on divergence level is only possible when there is a gap between intraspecies and interspecies divergence [50]. Low levels of *COI* divergences between congeneric species could reflect short histories of reproductive isolation [77]. In this case *COI* sequence divergence should not be used on its own but combined with additional data types using integrative taxonomy approach. Vujić et al. [10] pointed out that the low sequence divergence does not hamper the use of the informative nucleotide changes as supporting characters in taxonomy of European *Pipiza*. Among Syrphidae flies interspecies divergence level less than 2% were found for species of different genera, *Merodon*, *Pipiza*, *Cheilosia* (see [1, 10, 78]).

The members of the *M*. *atratus* species complex can be identified by the following morphological characters: tergites 2–4 completely with black pile or with stripes of black pile (Fig 4A–4C), while in the *M*. *cinereus* species complex tergites 2–3 only have pale pile (Fig 4D). Differences between *M*. *atratus* species and two other species from the complex are clear in tergites 3 and 4. The tergites 3 and 4 are completely covered with black hares in *M*. *atratus* while in *M*. *virgatus* **sp. nov.** and *M*. *balkanicus* **sp. nov.** there is stripe of pale hairs on tergite 3 and top of tergite 4. Morphological character states failed to distinguish between *M*. *virgatus* **sp. nov.** and *M*. *balkanicus* **sp. nov.** Subtle morphological differences together with molecular differentiation, wing and surstylus shape variations, and environmental niche analysis resulted in the separation of three species within the *M*. *atratus* species complex: *M*. *atratus*, *M*. *virgatus* **sp. nov.** and *M*. *balkanicus* **sp. nov.** The level of *COI* sequence divergence indicates a genetic separation that is concordant with geometric morphometric evidence. These species are allopatric and, thus, are exposed to different environmental backgrounds that could also explain the evolutionary divergence between them. Additionally, the distribution ranges of the taxa in the *M*. *atratus* species complex also suggest their separate evolution.

Morphologically indistinguishable *M*. *virgatus* **sp. nov.** and *M*. *balkanicus* **sp. nov.** are the most distinct species based on *COI* sequence divergence (p = 1.4%) and diverged from *M*. *atratus* (p distance values: 0.8% and 0.9%). The species do not share *COI* haplotypes which additionally confirmed their genetic separation. The lack of shared haplotypes indicates either strict reproductive isolation or ongoing selection against mitochondrial exchange between members of the complex [77]. The resolved species clusters and nodal support values in phylogenetic trees (MP; ML) also supported existence of three species within *M*. *atratus* species complex (Fig 6 and S1 Fig). Highly statistically significant divergences in wing and surstylus shape were found among all investigated species of the *M*. *atratus* species complex. In contrast to the molecular results, the highest distinctiveness in wing and surstylus shape was between the *M*. *atratus* and *M*. *balkanicus* **sp. nov.**, while *M*. *virgatus* **sp. nov.** and *M*. *balkanicus* **sp. nov.** were the most similar species. The results of Mantel test showed no association between wing/surstylus shape and geographic distribution of species.

Wing geometric morphometry has become a generally accepted and increasingly utilized methodology in insect and hoverfly taxonomic studies. It has shown great discriminatory power, even within closely-related taxa that are morphologically inseparable based on traditional methods [9, 10, 17, 18]. Due to the high heritability of insect wing shape, this significant separation is important evidence supporting species delimitation. In addition, the shapes of different structures of male genitalia constitute important characteristics for hoverfly taxonomy and systematics [79]. The structures of the male genitalia of species belonging to the *M*. *aureus* species group are extremely similar, and are virtually impossible to distinguish by means of traditional observation. Geometric morphometrics applied to male genitalia have proven to be a powerful tool in revealing subtle morphological variation, especially in closely-related insect taxa that are otherwise morphologically very similar [9, 17, 18, 80].

### Biogeographical Aspects

The distributional areas of the taxa belonging to the *Merodon atratus* species complex include high mountain ranges of central Europe (Alps) and the Balkan Peninsula. According to Schmitt [81], there is evidence of biogeographical connections between Alpine and western Balkan mountain systems, but based on our present results it seems that their physical separation is sufficient for speciation events to take place in syrphid flies. In contrast, eastern and western mountains of the Balkans are biogeographically disjunct [82], despite their physical closeness. The elevation ranges of the taxa in the *M*. *atratus* species complex range from around 1000 m to 2000 m for *M*. *balkanicus* **sp. nov.** and *M*. *virgatus* **sp. nov.** to 3000 m for *M*. *atratus*. This suggests spatial separation between their distributional areas and, thus, an island-shaped mountain range topology (Fig 3A and 3B) that could have influence on separate evolution of the taxa within this species complex. According to estimated divergence times, taxon splitting in the *M*. *atratus* species complex took place during the Pleistocene epoch. The taxa probably diverged during the interglacial periods between the Günz and Mindel glaciations (Mindel glaciations took place from 480 ka BP– 434 ka BP) and between the Ris and Mindel glaciations (434 ka BP—232 ka BP) [68, 69].

Ačanski et al. [18] discovered that the diversification processes of *Merodon avidus* species complex also took place during Pleistocene. *M*. *ibericus* Vujić, 2015 diverged from the rest of the complex during Calabrian stage of the Early Pleistocene (around 800 ky BP). *M*. *megavidus* Vujić and Radenković, 2016 separated from the *M*. *avidus* (Rossi, 1790)+*M*. *moenium* Wiedemann in Meigen, 1822 during Günz-Mindel interglacial and the diversification between *M*. *avidus* and *M*. *moenium* took place at the end of the Riss-Würm interglacial or the beginning of the Würm glaciation period [18]. Climatic and topological changes during glacial and interglacial periods had a strong influence on diversification processes in the European high mountain systems. As the *M*. *atratus* species complex comprises taxa with mountainous distributions that are better adapted to cold climates, we assume that during interglacial periods when climatic conditions changed, taxa shifted to higher elevations thus tracking their preferred habitats. Habitat fragmentation brought about by changing climate facilitated the independent evolution of taxa on mountain islands as we have shown in this study.

The impact of Pleistocene climate change on diversification processes is still not fully clear. Alternating glacial and interglacial periods during the Pleistocene resulted in population range expansions and contractions [83], contributing to the debate about the possibility of allopatric speciation. It is unclear whether a period of isolation in allopatry before the next range expansion was sufficient for speciation. Knowles [84] indicated that speciation does not need to be inhibited by glaciation, but can be restricted to a mode that is very rapid, such as speciation involving sexual selection or natural selection of ecologically-differentiated taxa. Additionally, recent studies have found that natural and sexual selection and their interaction also promote insect genital evolution [85, 86].

The taxa of the *M*. *atratus* species complex are all distributed on mountain ranges and although their preferred environmental niche space may seem similar, it is not identical. The low niche overlap values between *M*. *atratus*, *M*. *balkanicus* **sp. nov.** and *M*. *virgatus* **sp. nov.** reflect their different environmental limitations. Also, the niche identity test rejected the null hypotheses that the environmental niches of investigated species pairs are equivalent and clearly indicated that our studied species are ecologically divergent.

## Conclusions

Using an integrative approach, i.e. combining morphology, genetic data, geometric morphometry data and environmental niche comparisons, we have shown that all applied data and methods support the taxonomic status and relationships of taxa within the *Merodon atratus* species complex as described here. We conclude that the species within this complex originated during the Pleistocene and our results support the taxonomic divisions between species of the *M*. *atratus* species complex and indicate the importance of the multiple processes involved in speciation. Geographical separation on mountain islands, coupled with adaptation to specific environmental niche spaces, provided conditions for the independent evolution of three taxa within the *M*. *atratus* species complex, and subtle changes in surstylus shape indicate the influence of sexual selection. As the distributional areas of the taxa are mountain ranges with complex climatic and geological histories, not all of the questions about species origin are resolved, but we consider that the present study partially clarifies the pattern of diversification within the *M*. *atratus* species complex.

## Supporting Information

S1 Appendix Additional material. (DOCX)

S1 Fig ML tree of *Merodon atratus* species complex. (TIF)

S1 Table List of specimens used for molecular analyses and GenBank accession numbers for obtained sequences. (XLSX)

S2 Table Haplotypes of combined *COI* sequences of *Merodon atratus* species complex. (XLSX)

S3 Table List of specimens used for wing geometric morphometric analysis, by geographical area and species. (XLSX)

S4 Table List of male specimens used for surstylus geometric morphometric analysis, by geographical area and species. (XLSX)

S5 Table Results of PCA and ANOVA. (XLSX)
