# Supplementary material for: Molecular and Morphological Inference of Three Cryptic Species within the Merodon aureus Species Group (Diptera: Syrphidae)
Source: PLoS One. 2016 Aug 17;11(8):e0160001. doi: 10.1371/journal.pone.0160001 (PMC4988715; doi:10.1371/journal.pone.0160001)
Supplement: S1 Appendix — (DOCX) [file pone.0160001.s001.docx]

**S1 Appendix**. **Additional material.**

*Merodon atratus* (Oldenberg 1919) **stat. nov.**

**Additional material.** AUSTRIA: ♂, Alps, Lech, (NHMW 01988); Carinthia: ♂, Amberg, 15.6.1973., (RMNH); ♂, Saualpe mountains, (ZHMB); Hohe Tauern: 18♀♀, 12♂♂, 10.8.2011. (FSUNS M89; N02; N03; N09; N14; N15; N16; N17; N18; N19; N20; N21; N27; N28; N38; N40; N41; N43; N44; N53; N55; N60; N61; N62; N64; N65; N67; N68; N72; N75); 1♂, Grossglockner, (MZH); 1♀, Kals, 10.8.2011., (FSUNS N74); ♀, Nussing, 10.8.2011., (FSUNS N77); ♀, Styria, 14.8.1990., (ZHMB); Tyrol: 4♀♀, 2♂♂, Dorfertal, N from Kals, 30.7.1973., (RMNH); 2♂♂, Lechtal beach, 29.7.1990., (RMNH); 1♂, Obergurgl, 02-03.8.1972. (RMNH); 2♂♂, Obertilliach, (MZH); 2♂♂, Ötztal Vent, 02.8.1967., (RMNH); 1♂, Seefeld Rosshütte, 15.7.1969., (RMNH); 1♀, Tauerntal, Matreier Tauernhaus, 31.7.1973., (RMNH); Vorarlberg:1♂, 03.7.1937., (NHMW); 1♂ Gargellen, (MZH); Hoher Freschen: 1♂,. 17.7.1936., (NHMW); 1♂26.7.1949., (NHMW); FRANCE: Provence Alpes Cote d'Azur: 1♂, Col du Lautaret, (ZHMB); 1♂, Le Lautaret, 02.8.1921., (MNHN); 1♂, La Grave, (MNHN); 1 ♂ Rhone Alpes, Abondance (He Savoie), 1929., (MNHN); Italy: Tirol: 2♂♂, 19.7.1984., (RMNH); 1♂, Stilfser Joch, 7.1871., (NHMW); 1♀, Trafoi, a village near Stelvio Pass (Scherfling, Pokorny), 12.8.09., (ZHMB); 1♂, Bozen, Seiser Alpe (Seiser Alm, Alpe di Siusi), (NHMW); SWITZERLAND: 4♂♂, below Riffelalp, 08.8.07., (LONDON DARWIN CENTER); Engadin: 1♂, Bergün, 12.7.1968., (RMNH); Preda: 2♂♂, 08.7.1968., (RMNH); 2♂♂, 11.7.1974., (RMNH); 1♂, 14.7.1974., (RMNH); Spinas: 31♂♂, 10.7.1968., (RMNH); 16♂♂, 10.8.1968. (RMNH); Graubünden: 1♂, Clavadel, 11.7.1974., (RMNH); Davos: 1♂, 05.7.1979., (RMNH); 2♂♂, 24-25.7.1979., (RMNH); 2♂♂, Pontresina, 7.1909. (ZHMB); 14♂♂, 8-14.7.1974., (RMNH); St Moritz: 2♂♂, 01-07.8.1974., (RMNH); 1♂, 16.7.1964., (RMNH); 5♂♂, 17.7.1960., (RMNH); 1♀, 17.7.1902., (SMNS 01973); 3♀♀, 20-22.7.1902., (SMNS 01967; 01971; 01972); 1♂, 24.7.1914., (ZHMB); 1♀, 22.7.1902., (ZHMB); 3♀♀, 2♂♂, (ZHMB); 3♂♂, Fexstal, 17.7.1960., (RMNH); 1♂, Sella Joch, Dolom. Mannhems, 09.71958., (RMNH); 2♂♂, Tirol Alm, 19.7.1984., (RMNH); 1♂, Uri-Andermatt, (MZH); 1♀, Valais-Zermatt, Sunnegga, 27.7.1984., (RMNH); 1♂, Vallistorbel-mosalp, 20.7.53., (MZH); 2♂♂, Wallis, Ritzingen, 31.7.1985. (RMNH).

*Merodon virgatus* Vujić et Radenković **sp. nov.**

**Paratypes.** BOSNIA AND HERZEGOVINA: 2♂♂, “Simony Bosnien” (NHMW 01982, 03885); CROATIA: 7♂♂, Velebit, Štirovača, 22.7.1967. leg. H.J.P Lambeck (RMNH); 1♂ Šatorina, 15.6.1910. leg. Meusel (FSUNS 04488); FRY Macedonia: 1♂ Šar-planina, Tearce, 22.7.1958 (FSUNS 01986); GREECE: 1♂, 7♀♀, Olympus, 10.8.2013. leg. J. Devalez; 7♀♀, Olympus, Petrostrouga, 2♂♂, Olympus, Petrostrouga, 13-20.7.2013. leg. K. Minachilis, 10-17.8.2013. leg. K. Minachilis; (all in MAegean); MONTENEGRO: Durmitor: 4♀♀, 8♂♂, 20-22.7.1997. leg. A. Vujić (FSUNS 01027; 01052; 01056; 01063; 01028; 01053; 01057; 01058; 01059; 01060; 01061; 01062); 4♀♀, 14♂♂, 30.07-02.8.1994. (FSUNS 01009; 01011; 01012; 01023; 01006; 01007; 01008; 01010; 01013; 01014; 01015; 01016; 01017; 01018; 01019; 01020; 01021; 01022); 8♀♀, 4♂♂, “dolina Škrčkih jezera”, 23.08.1994. (FSUNS 00952; 00961; 00962; 00963; 00964; 00965; 00966; 00967; 00968; 00969; 00980; 01081); 4♀♀, 2♂♂, Jablan bara, 12.8.1984 (FSUNS 0956; 00957; 00958; 00959; 00960; 00970); Jablan jezero 1♀, 10.8.2013. leg. A. Vujić (FSUNS AE5); 2♀♀, 5♂♂, 31.7.1995. (FSUNS 00953; 00954; 01002 01003; 01004; 01005; 01079); Sušica River Canyon: 1♀, 03.8.1982. (FSUNS 00982); 3♀♀, 1♂, 24.8.1984. (FSUNS 00950; 00979; 00981; 01043); 2♀♀, 31.7.1998. (FSUNS 01037; 01038); 1♂, Tara River Canyon, 25.7.1981. (FSUSN 00987); Krecmani: 1♀, 1♂, 26.8.1999. (FSUNS 01050; 01051); 7♀♀, 30.7.1998. (FSUNS 01035; 01036; 01039; 01040; 01041; 01921; 01923); 2♀♀, Luke Skakala, 24.8.1984. (FSUNS 00977; 00978); 1♂, “Mali Međed”, 13.8.1984. (NBCN); 1♀, Milnski potok, 06.8.1985. (FSUNS 00986); 7♂♂, Prutaš, 27.7.2011. (FSUSN M73; M75; M76; M77; M78; M79); “Savink kuk” 6♀♀, 5♂♂, 01.8.1998. (FSUSN 01029; 01030; 01032; 01033; 01049; 01924; 01031; 01034; 01069; 01070; 01922); 24♀♀, 3♂♂, 28.8.2011.leg. A. Vujić, (FSUNS S45-S73); 3♀♀, “Savina voda”, 28.7.1998. leg. A.Vujić, (FSUNS 01042; 01044; 01925); 10♀♀, Sedlo, 04.8.1982. leg. A. Vujić (00949; 00995; 00996; 00997; 00998; 00999; 01000; 01001; 01080; 01926); 4♀♀, Stožina, 04.8.1982. leg. A. Vujić (FSUNS 00991-00994); 1♂, Surutke, 28.8.1984. (FSUSN 00983); 2♀♀, Sušićko jezero, Sastavci, 25-26.6.1997. FSUSN (01054; 01055); Škrčka jezera: 7♀♀, 1♂, 09.8.1985. leg. A. Vujić (FSUNS 00951; 00955; 00971; 00972; 00973; 00974; 00975; 00976); 1♂, 17.7.1997. leg. A. Vujić (FSUNS 01078); 1♂, 23.7.2009. leg. A. Vujić (FSUNS B20); 1♀, 23.8.1984. (FSUNS); “Škrčko ždrijelo”: 2♀♀, 3♂♂, 02.9.1997. leg. A. Vujić (FSUNS 01064-01068); 2♀♀, 8.1997. leg. A. Vujić (FSUSN 01047; 01048); 6♂♂, 17.7.1997. leg. A. Vujić (FSUSN 01045; 01046; 01071-01073; 01920); 12♀♀, 19♂♂, 27.7.2011. leg. A. Vujić (FSUNS M42-M72); 3♀♀, 1♂, Krecmani, 30.7.1998. leg. A. Vujić (FSUNS 01074-01077); 1♀, 2♂♂, “Velike Lokvice”, 06.8.1996. leg. A. Vujić, (FSUNS 01024-01026); 3♀♀, “Veliki Štuoc”, 02.8.1982. leg. A. Vujić, (FSUSN 00988-00990); 1♀, 1♂, “Zeleni vir”, 26.8.1984. leg. A. Vujić, (FSUNS 00984; 00985); 2♂♂; Prokletije, 26-30.7.1994. leg. A. Vujić, (FSUNS 01927-01928); SERBIA: Šar-planina: 1♀, 1♂, Berevački potok, 16-20.7.1995. leg. A. Vujić, (FSUNS 02929-01930); 2♀♀, Grulov potok, 04.8.1991. leg. A. Vujić, (FSUNS 01929-01930); 2♀♀, Grulov potok, leg. A. Vujić, (FSUNS 01931; 01932); 1♀, 1♂, Muržica, 12.7.1996. leg. A. Vujić, (FSUNS 01933; 01934).

*Merodon balkanicus* Šašić, Ačanski et Vujić **sp. nov.**

**Paratypes.** SERBIA: 7♂♂, 3♀♀, Stara Planina, “Babin zub”, 11.7.2011., leg. Vujić (FSUNS L95; L98; L99; M1; M2; M5; M6; M7; M8; M10); 2♀♀ Stara Planina, above Arbinje, 26.8.2013., leg. Vujić; BULGARIA: 1♂ Rhodopi, Belmeken, 01.7.1912., leg. Drensky (NMNHS 05869).
