# Supplementary figures and images for: Molecular and Morphological Inference of Three Cryptic Species within the Merodon aureus Species Group (Diptera: Syrphidae)
Source: PLoS One. 2016 Aug 17;11(8):e0160001. doi: 10.1371/journal.pone.0160001 (PMC4988715; doi:10.1371/journal.pone.0160001)

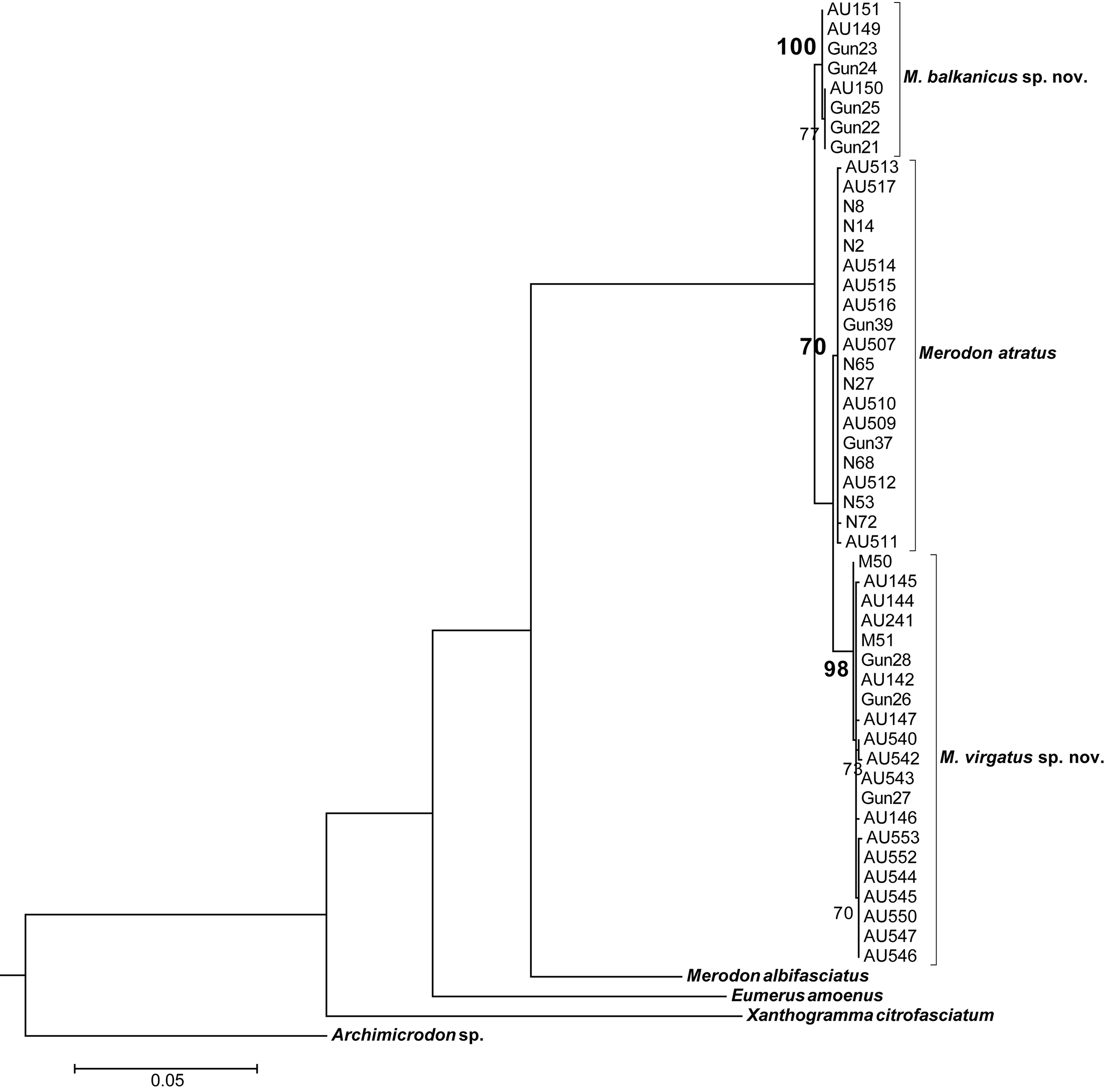

Supplement: S1 Fig — (TIF) [file pone.0160001.s002.tif]
